# The Secreted Peptide PIP1 Amplifies Immunity through Receptor-Like Kinase 7

**DOI:** 10.1371/journal.ppat.1004331

**Published:** 2014-09-04

**Authors:** Shuguo Hou, Xin Wang, Donghua Chen, Xue Yang, Mei Wang, David Turrà, Antonio Di Pietro, Wei Zhang

**Affiliations:** 1 School of Life Science, Shandong University, Jinan, Shandong, China; 2 School of Municipal and Environmental Engineering, Shandong Jianzhu University, Ligang Developmental Zone, Jinan, Shandong, China; 3 College of Technological Gardening, Shandong Yingcai University, Jinan, Shandong, China; 4 Departamento de Genética, Universidad de Córdoba, Córdoba, Spain; Oregon State University, United States of America

## Abstract

In plants, innate immune responses are initiated by plasma membrane-located pattern recognition receptors (PRRs) upon recognition of elicitors, including exogenous pathogen-associated molecular patterns (PAMPs) and endogenous damage-associated molecular patterns (DAMPs). *Arabidopsis thaliana* produces more than 1000 secreted peptide candidates, but it has yet to be established whether any of these act as elicitors. Here we identified an *A. thaliana* gene family encoding precursors of PAMP-induced secreted peptides (prePIPs) through an *in-silico* approach. The expression of some members of the family, including *prePIP1* and *prePIP2*, is induced by a variety of pathogens and elicitors. Subcellular localization and proteolytic processing analyses demonstrated that the *prePIP1* product is secreted into extracellular spaces where it is cleaved at the C-terminus. Overexpression of *prePIP1* and *prePIP2*, or exogenous application of PIP1 and PIP2 synthetic peptides corresponding to the C-terminal conserved regions in prePIP1 and prePIP2, enhanced immune responses and pathogen resistance in *A. thaliana*. Genetic and biochemical analyses suggested that the receptor-like kinase 7 (RLK7) functions as a receptor of PIP1. Once perceived by RLK7, PIP1 initiates overlapping and distinct immune signaling responses together with the DAMP PEP1. PIP1 and PEP1 cooperate in amplifying the immune responses triggered by the PAMP flagellin. Collectively, these studies provide significant insights into immune modulation by *Arabidopsis* endogenous secreted peptides.

## Author Summary

Both animals and plants have evolved mechanisms to trigger innate immunity through perception of exogenous and endogenous molecules. In the model plant *Arabidopsis thaliana*, endogenous molecules such as the peptide elicitor PEP1 activate the immune response by means of cell surface-located receptors. Here we describe a new gene family in *A. thaliana* named *prePIPs*, whose members encode secreted peptide precursors, and show that one of its members, prePIP1, is secreted into extracellular space and cleaved at the C-terminus. Exogenous application of PIP1, the synthetic 13-amino acid peptide corresponding to the conserved C-terminal region of prePIP1, triggered immune responses and led to enhanced pathogen resistance in *A. thaliana*. We further provide evidence showing that PIP1 signals via the receptor-like kinase 7 (RLK7) and employs both shared and distinct components with the PEP1 signaling pathway. Both PIP1 and PEP1 cooperatively amplify the immune response triggered by flg22, the active epitope of bacterial flagellin.

## Introduction

Immune signaling in plants is typically initiated when immune-related receptors perceive the presence of pathogen molecules, including so-called “pathogen-associated molecular patterns” (PAMPs) and race-specific effectors [1]. PAMPs, such as bacterial flagellin and fungal chitin, are recognized by plasma membrane-located pattern recognition receptors (PRRs), which activate PAMP-triggered immunity (PTI). In addition, pathogen infection causes the release of endogenous damage-associated molecular patterns (DAMPs), such as peptides, oligogalacturonides (OGs), or cutin monomers. DAMPs are released from the cytoplasm or the cell wall into the extracellular space, where they induce immune responses resembling PTI following perception by PRRs [2]–[4]. Over a dozen PRRs have been identified. Most belong to the superfamily of receptor-like kinases (RLKs), characterized by an extracellular domain, a transmembrane region and a cytoplasmic kinase domain. *Arabidopsis thaliana* has more than 600 RLKs. Among these, the leucine-rich repeat RLKs (LRR-RLKs) constitute the largest group which has been divided into 13 categories (I through XIII) [5]. Flagellin-sensitive 2 (FLS2), a LRR-RLK from category XII, binds to a 22 residue epitope (flg22) present at the N terminus of flagellin from Gram-negative bacteria [6]. Perception of flg22 induces the dimerization and rapid phosphorylation of FLS2 and BRASSINOSTEROID INSENSITIVE 1-associated receptor kinase 1 (BAK1), as well as phosphorylation of the receptor-like cytoplasmic kinase (RLCKs) BIK1 [7]–[10]. The activated receptor complex triggers elevation of cytosolic calcium, generation of reactive oxygen species (ROS), phosphorylation of mitogen-activated protein kinases (MAPKs), callose deposition, and transcriptional reprogramming of the cell, leading to enhanced resistance against pathogens [11]–[14].

PEP1 was identified as an extracellular 23-aa peptide derived from the C-terminus of the *A. thaliana* precursor protein proPEP1. Since proPEP1 lacks an N-terminal signal peptide, the release of PEP1 into the apoplast was suggested to result from cellular damage caused by pathogen attack or wounding, suggesting that PEP1 functions as DAMP. Two XI category LRR-RLKs, PEPR1 and PEPR2, were shown to act as receptors of PEP1 and homologous peptides in *A. thaliana* [15], [16]. Perception of PEP1 by PEPR1/2 activates PTI and enhances host resistance against the pathogens *Pseudomonas syringae* and *Pythium irregulare* [3], [16]. PEPR1 also modulates ethylene (ET)-dependent resistance to *Botrytis cinerea* via the phosphorylation of BIK1 [17], [18]. Since expression of PEP1-PEPR1/2 is induced by flg22 and PEP1 itself, and since PEP1-PEPR1/2 employs shared components with PAMPs signaling, PEP1-PEPR1/2 has been proposed to function as an amplifier of PTI signaling [16], [19].

Secreted peptides coordinate a variety of plant developmental processes, including stem cell maintenance, stomatal development, lateral root initiation, vascular formation, floral abscission and cell expansion [20]–[22]. Recently, several secreted peptides have been reported to modulate plant immune signaling. For instance, the CLAVATA3 peptide (CLV3p), known to regulate stem cell homeostasis in the shoot apical meristem, was suggested to be recognized by FLS2 and activate FLS2-dependent immune responses in the shoot meristem [23]. The sulfated peptides phytosulfokine (PSK) and PSY1, were initially identified as promoters of cell proliferation and tissue growth, and were recently shown to attenuate PTI responses and to enhance susceptibility to biotrophic pathogen and resistance to necrotrophic pathogen [24], [25]. *A. thaliana* has been suggested to produce over 1000 secreted peptides [26], the overwhelming majority of which remain functionally uncharacterized. To look for secreted peptides potentially involved in regulation of immunity, we searched the available *A. thaliana* microarray data for flg22- and elf18-induced genes. This led to the identification of a novel gene family of secreted peptide precursors, termed “*prePIPs*” (precursors of PAMP-Induced Peptides). We provide evidence showing that PIP1 and PIP2, two peptides obtained from processing of the representative *prePIP* family members *prePIP1* and *prePIP2*, are able to activate immune responses in *A. thaliana* and to enhance resistance against *P. syringae* and *Fusarium oxysporum*. Using a reverse genetics approach, we demonstrate that RLK7, a class XI LRR-RLK, is required for PIP1 and PIP2-elicited immune activation, and that PIP1-RLK7 has a crucial role in PTI amplification.

## Results

### Screening of *A. thaliana* genes encoding PAMP-induced secreted peptide (PIP) precursors

**Figure 1:**
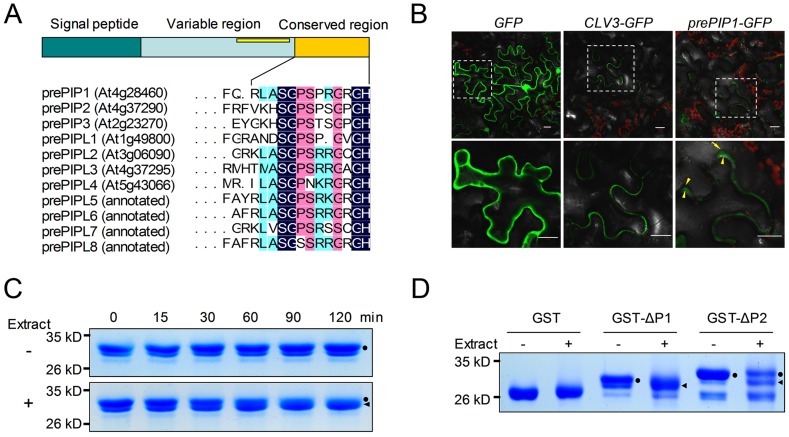
Identification of PIP peptides. (A) Schematic presentation of *prePIP* homologs in *A. thaliana*. (B) Sub-cellular distribution of prePIP1-GFP in tobacco leaf cells. Tobacco leaves were transformed with *Agrobacterium* GV3101 harboring a construct containing *GFP*, *prePIP1-GFP* or *CLV3-GFP*, respectively. The yellow arrows point the plasma member. Scale bar = 20 µm. (C) Time-course of GST-ΔP1 proteolytic processing. (D) Proteolytic cleavage of GST-ΔP1 and GST-ΔP2 by total protein extract from *A. thaliana*. (C–D) SDS-PAGE separation of protein products. Dots mark intact GST-ΔP1 or GST-ΔP2; triangles mark processed GST-ΔP1 or GST-ΔP2. At least three replicates were performed with similar results.

Analysis of flg22- and elf18-induced transcription data (microarray accession number E-MEXP-547) resulted in the identification of 12 genes encoding putative secreted peptide precursors [27]. The predicted gene products were 70–110 amino acid residues in length and included an N terminal signal peptide, as predicted by the SignalP 3.0 server [28]. Of these, four have known or predicted functions. They include PSK4 precursor [29], PSY1 precursor [30], IDA [31], and an IDA-like protein (At1g05300). The other eight are functionally uncharacterized. Three of these eight genes (At4g28460, At4g37290, and At2g23270) share a highly conserved C terminus, and their products were named prePIP1, prePIP2 and prePIP3, respectively (Table S1). A blastp search based on the prePIP1 C terminus sequence revealed that *A. thaliana* has at least 11 prePIP homologs, including seven annotated and four non-annotated proteins. Orthologs of prePIP proteins are present in numerous species of dicots and monocots, such as soybean, grape, maize, and rice (Figure S1). All the prePIP family members exhibit the hallmarks of post-translationally modified secreted peptide precursors: a signal peptide at the N terminus, a highly conserved cysteine-poor region at the C-terminus (hereafter referred to as the SGPS motif), and a variable region between the signal peptide and the SGPS motif (Figure 1A) [20]. Eight *A. thaliana* family members contain a single SGPS motif while three (prePIP2, prePIP3 and prePIPL1) harbor two SGPS motifs. The prePIP SGPS motif shares structural features with CLV3/CLE peptides [32], [33], the IDA peptide (IDAp), CEP1 [34], and PEP1 [3]. Since all these peptides carry conserved Ser, Gly, Pro, and His residues (Figure S2), we propose that they form a superfamily called “SGP-rich” peptides.

During the process of translation, the prepropeptide, the original form of secreted peptide precursor, is targeted to the endoplasmic reticulum/Golgi-dependent secretory pathway where the N-terminal signal peptide is removed resulting in the propeptide. The propeptide is subsequently secreted into the apoplast and subjected to proteolytic processing, releasing the mature C-terminal peptide [20]. To experimentally determine whether the prePIP1 propeptide is secreted, the green fluorescent protein gene (*GFP*) was fused to the C-terminus of *prePIP1* (*prePIP1-GFP*) under the control of the cauliflower mosaic virus 35S (CaMV 35S) promoter and transiently expressed in tobacco leaves using agro-infiltration. Confocal microscopy imaging showed that prePIP1-GFP fluorescence was distributed in the pericellular apoplastic space. In contrast, GFP protein alone was present in the cytoplasm and the nucleus. The secreted peptide precursor CLV3, which was previously shown to localize in the extracellular matrix, exhibited a similar localization as prePIP1-GFP when a C-terminal *GFP* fusion allele was expressed in tobacco leaves (Figure 1B). These results suggest that the *prePIP1* product is secreted into the plant extracellular space.

An *in-vitro* assay was conducted to determine whether prePIP1 and prePIP2 are proteolytically processed. Glutathione S-transferase-tagged signal peptide-deleted prePIP1 and prePIP2 (GST-ΔP1 and GST-ΔP2) were expressed in *E. coli* strain BL21 (DE3) and purified through Glutathione Sepharose (Figure S3). Incubation of GST-ΔP1 and GST-ΔP2 in a reaction solution supplementing extracts of *A. thaliana* seedlings but not BSA (negative control) resulted in a reduction of 1–2 kDa in size (Figure 1C and D). When GST-ΔP1 was injected into *A. thaliana* leaves, a similar reduction in molecular size was detectable after a 2 h incubation, consistent with a cleavage of GST-ΔP1 by a plant protease(s) present in the extracellular space (Figure S4).

### Expression of prePIP1

**Figure 2:**
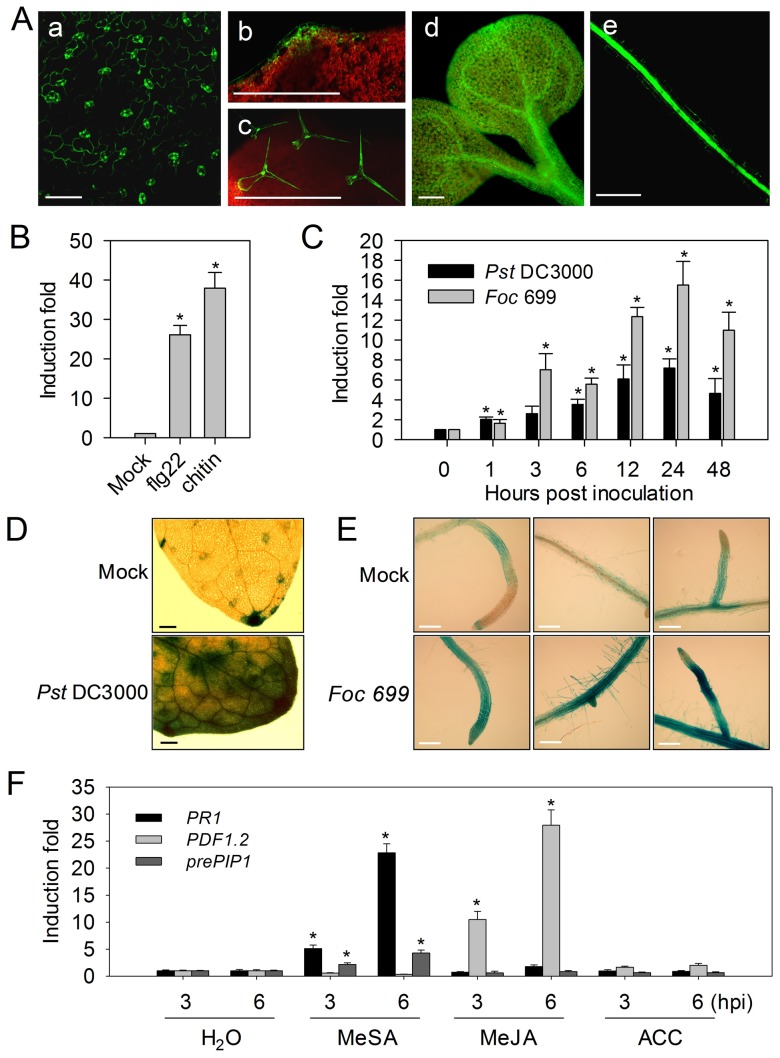
Expression of prePIP1. (A) Transgenic *A. thaliana* expressing *GFP* driven by the *prePIP1* promoter in (a) the guard cell, (b) the hydathode, (c) the epidermal trichome, (d) the leaf vascular tissue and (e) the root vascular tissue. (B) RT-qPCR-based transcriptional profiling of *prePIP1* in *A. thaliana* following treatment with flg22 or chitin. (C) RT-qPCR-based transcriptional profiling of *prePIP1* in *A. thaliana* following inoculation with *Pst* DC3000 or *Foc* 699. GUS staining of *prePIP1p-GUS* transgenic *A. thaliana* seedlings after a 24 h exposure to *Pst* DC3000 (D), and *Foc* 699 (E). Scale bar = 200 µm. (F) RT-qPCR-based transcriptional profiling of *prePIP1*, *PR1*, and *PDF1.2* in *A. thaliana* following exposure to MeSA, MeJA, and ACC. Error bars represent ± standard error (SE) of the mean (n = 3). *: difference significant at p<0.01 (*t*-test). Three replicates were performed with similar results.

Transgenic plants carrying the *GFP* gene under control of the *prePIP1* promoter exhibited strong fluorescence in guard cells, hydathodes and vascular tissue (Figure 2A). Interestingly, all these tissues represent either potential entry points or proliferation routes for invading pathogens. In contrast, no fluorescence was detected in these tissues in untransformed plants (data not shown). When *A. thaliana* seedlings were exposed to flg22 or chitin, *prePIP1* transcription was markedly up-regulated (Figure 2B). Subsequent experiments, based either on transcript abundance or on the expression of a transgene carrying the β-glucuronidase (*GUS*) gene driven by the *prePIP1* promoter, confirmed that *prePIP1* was up-regulated during infection with the bacterial pathogen *P. syringae* DC3000 (*Pst* DC3000) or with the fungal pathogen *F. oxysporum* f. sp. *conglutinans* strain 699 (*Foc* 699) (Figure 2C). Transcript abundance increased about eight folds following inoculation with *Pst* DC3000 and about 15 folds with *Foc* 699, extending throughout the leaf and root system within 24 h after inoculation (Figure 2C–E). *PrePIP1* expression was also increased in *A. thaliana* seedlings after treatment with immune-related phytohormones. Quantitative RT-PCR (RT-qPCR) analysis showed that the *prePIP1* transcript was induced by methyl salicylate (MeSA), but not by methyl jasmonate (MeJA) or the ethylene precursor 1-aminocyclopropane-1-carboxylate (ACC). Importantly, expression of the SA pathway marker pathogenesis-related protein 1 (*PR1*) and of the JA pathway marker *PDF1.2* was induced by MeSA and MeJA treatments, respectively (Figure 2F).

### PIP1 and PIP2 inhibit *A. thaliana* root growth

**Figure 3:**
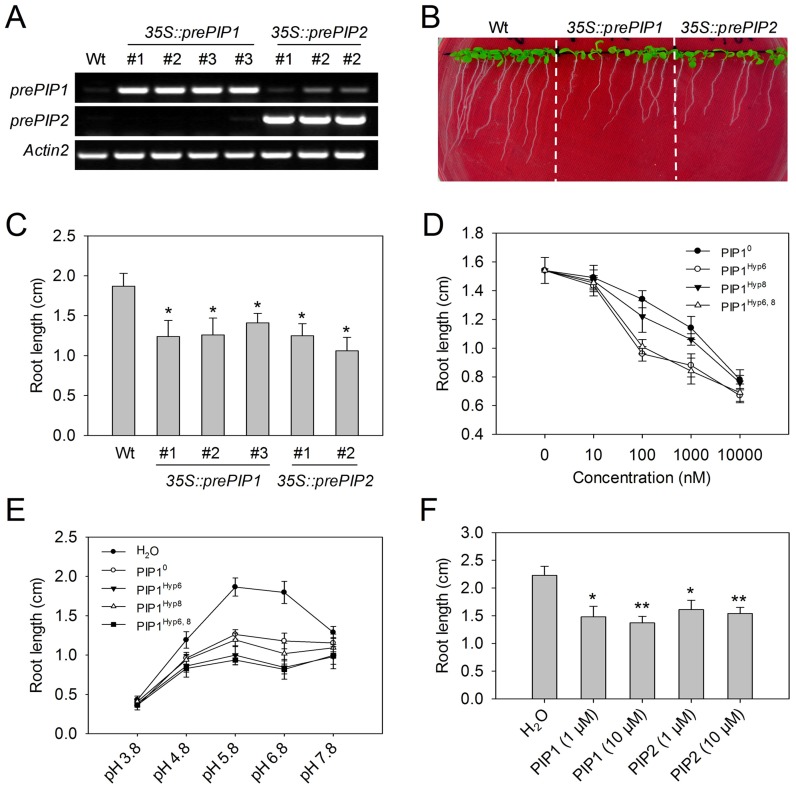
Root growth is inhibited by PIP1 and PIP2. (A) RT-PCR-based detection of *prePIP1* and *prePIP2* transcripts in transgenic *A. thaliana*. (B) Morphology and (C) root length of eight day old WT, *35S::prePIP1* and *35S::prePIP2* transgenic seedlings. (D) Effect of the concentration of PIP1 derivatives on *A. thaliana* root growth inhibition. (E) Effect of pH on PIP1-induced root growth inhibition. (F) *A. thaliana* root growth is inhibited by PIP1 and PIP2. Error bars represent the SE of the mean (n>30), *, **: differences significant at p<0.01, 0.001 (*t*-test). Three replicates were performed with similar results.

*A. thaliana* transgenic lines overexpressing *prePIP1* or *prePIP2* (*35S::prePIP1* and *35S::prePIP2*) (Figure 3A) consistently exhibited a shorter main root than the wild type (WT) plants (Figure 3B and C). In contrast, transgenic plants overexpressing IDA and IDA-like were abnormal with respect to their floral abscission zone (AZ) [35]. In spite of the high sequence similarity between the C termini of IDA and prePIPs, overexpression of prePIP1 or prePIP2 did not affect AZ structure (Figure S5), indicating different functions of the two protein families.

Because post-translationally modified secreted peptides generally coincide with the C-terminal conserved region of their precursors [20], exogenous application of synthetic peptides such as CLV3p, IDAp, and CEP1 reproduces the phenotypes of overexpression lines of the respective precursor gene [32], [34], [35]. We tested whether addition of synthetic peptide PIP1^0^ comprising the conserved SGPS-motif of prePIP1, could reproduce the effect on root growth of prePIP1 overexpression. PIP1^0^ significantly inhibited the elongation of the main root when applied at a concentration of 100 nM (Figure 3D). Since SGP-rich peptides usually undergo proline hydroxylation, the inhibitory effect on root growth of three PIP1 derivatives, PIP1^Hpy6^, PIP1^Hpy8^, and PIP1^Hpy6, 8^ (Table S2) was investigated. Of these, PIP1^Hyp6^ (hereafter denoted “PIP1”) and PIP1^Hpy6, 8^ were more active than PIP1^0^ (Figure 3D), suggesting that proline hydroxylation at position 6 contributes to biological activity of the peptide. PIP1 activity was also pH dependent, since root growth inhibition was most active in the pH range 5.8–6.8 (Figure 3E). Results for PIP2, the synthetic hydroxylated peptide corresponding to prePIP2, were similar to those obtained with PIP1 (Figure 3F).

### PIP1 and PIP2 elicit immune responses in *A. thaliana*

**Figure 4:**
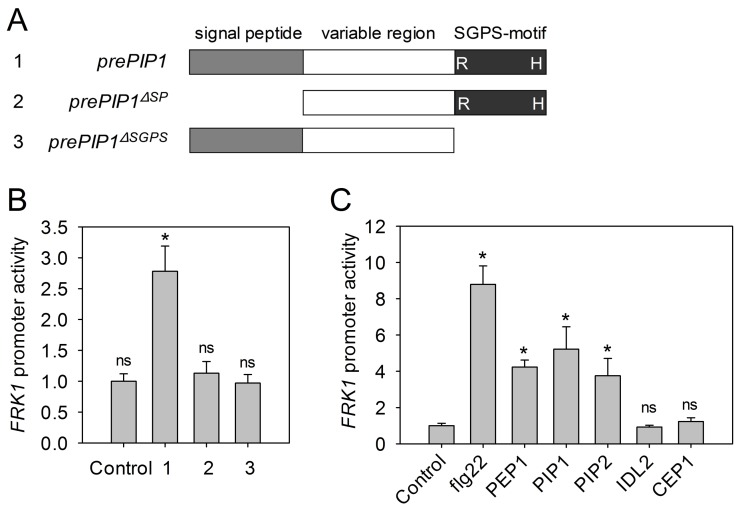
The *FRK1* promoter is activated by PIP1 and PIP2. (A) Schematic presentation of the constructs containing *prePIP1* and truncated *prePIP1* sequences. (B) *FRK1* promoter activation in protoplasts following co-transfection with *FRK1p-LUC* and *prePIP1* or truncated *prePIP1*. (C) *FRK1* promoter activation by PIP1, PIP2, flg22, and PEP1. Protoplasts transfected with *FRK1p-LUC* were exposed to 1 µM of each peptide for 4 h. (B–C) Error bars represent the SE of the mean (n = 5), *: significantly different from control at p<0.01 (*t*-test), ns: non significant difference. Three replicates were performed with similar results.

**Figure 5:**
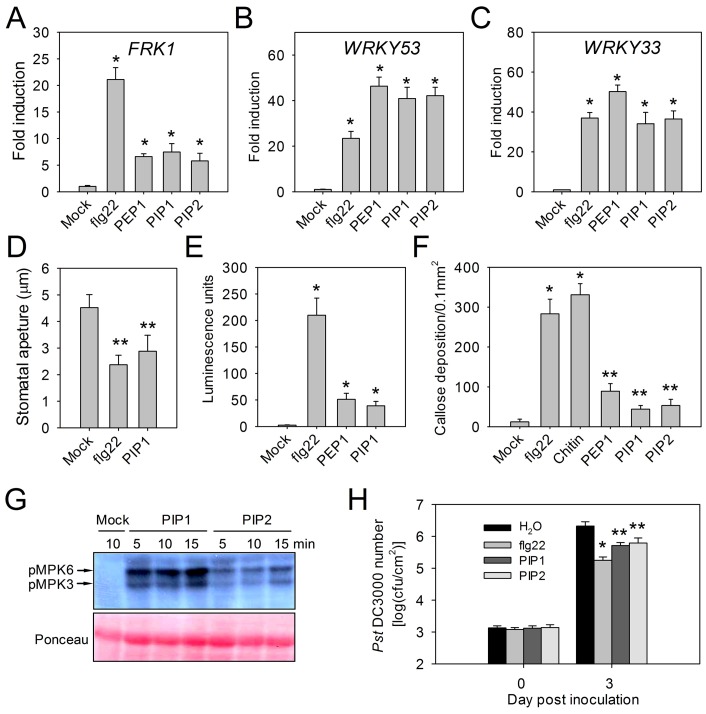
Immune response activation by PIP1 and PIP2. Transcription of (A) *FRK1*, (B) *WRKY53*, (C) *WRKY33* in *A. thaliana* seedlings treated with flg22, PEP1, PIP1, and PIP2. Error bars represent the SE of the mean (n = 3). At least three replicates were performed with similar results. (D) Stomatal closure induced by PIP1 and flg22. Error bars represent the SE of the mean (n>100). Three replicates were performed with similar results. (E) Relative ROS production in adult leaves upon treatments with PIP1, PEP1, and flg22. Error bars represent the SE of the mean (n = 5). Two replicates were performed with similar results. (F) Callose deposition in leaves upon induction with different peptides or chitin. Error bars represent the SE of the mean (n = 5). Two replicates were performed with similar results. (G) MAPK activation induced by PIP1 and PIP2. Ten day old seedlings were exposed to 1 µM peptides for 5, 10 or 15 min. Western blot analysis was performed with the phospho-p44/42 MAPK antibody. Two replicates were performed with similar results. (H) *Pst* DC3000 growth in *A. thaliana* leaves. Error bars represent the SE of the mean (n = 6). *, **: significantly different from mock treatment at p<0.001 and <0.01 (*t*-test). Three replicates were performed with similar results.

The role of the peptide derived from prePIP1 in plant immunity was explored initially using a transient expression assay in mesophyll protoplasts. The firefly luciferase gene (*LUC*) driven by the promoter of *Flg22-induced Receptor-like Kinase 1* (*FRK1*), a marker gene of PTI signaling, was co-transfected as a reporter with either *prePIP1*, *prePIP1^ΔSP^* (*prePIP1* lacking the signal peptide), or *prePIP1^ΔSGPS^* (*prePIP1* lacking the SGPS-motif) all driven by the CaMV 35S promoter. Activation of the *FRK1* promoter was only detected with a full length copy of *prePIP1*, implying that both secretion of prePIP1 and its SGPS-motif are required for *FRK1* induction (Figure 4A and B). Similar to flg22 and PEP1, exogenous application of PIP1 and PIP2 induced the expression of *pFRK1::LUC* in protoplasts, but neither IDL2 nor CEP1 did (Figure 4C). Moreover, RT-PCR and RT-qPCR analyses revealed that PIP1 and PIP2 induced transcription of the immune response genes *FRK1*, *WRKY30*, *WRKY33*, *WRKY53*, and *PR1* (Figure 5A–C and S6). Other characteristic PTI responses such as stomatal closure (Figure 5D), ROS production (Figure 5E), callose deposition (Figure 5F), and MAPK phosphorylation (Figure 5G) were also induced by these two peptides. In comparison with flg22, PIP1, PIP2 and PEP1 induced significantly lower ROS production and leaf callose deposition (Figure 5E and F). Similarly, the effect of PIP-induced immunity on host resistance against *Pst* DC3000 was weaker than that induced by flg22. Treatment with 1 µM PIP1 or PIP2 delayed *Pst* DC3000 proliferation in leaves by ∼70%, while 1 µM flg22 decreased bacterial growth by >90% (Figure 5H).

**Figure 6:**
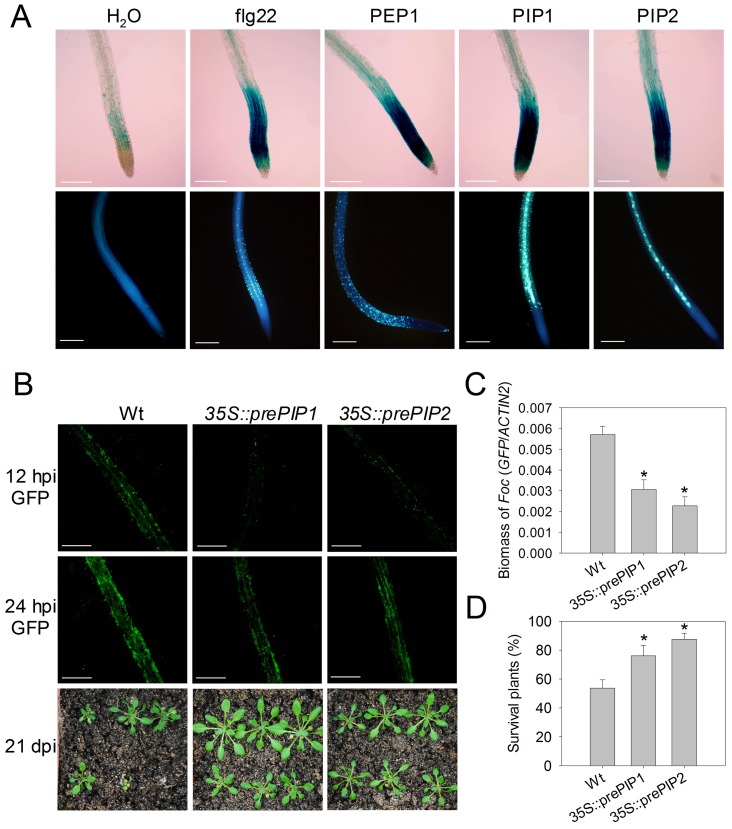
Immune response activation in roots by PIP1 and PIP2. (A) *MYB51p::GUS* expression (top panel) and callose deposition (lower panel) in *A. thaliana* seedlings exposed to peptide elicitors. Two replicates were performed with similar results. (B) *Foc* 699-*GFP* infection in WT, *35S::prePIP1* and *35S::prePIP2* seedlings. Top and center: GFP signal in roots of *A. thaliana* seedlings after 12–24 hour' infection with *Foc* 699-*GFP* (scale bar = 0.5 mm). Bottom: representative plants 21 days post infection. Three replicates were performed with similar results. (C) Quantification of fungal biomass in *35S::prePIP1* and *35S::prePIP2* transgenic seedlings 12 h after infection with *Foc* 699-*GFP*. (D) Survival of plants 21 days after infection with *Foc* 699-*GFP*. (C–D) Error bars represent the SE of three replicates that contained 30 to 40 plants or seedlings each. *: significantly different from control at p<0.01 (*t*-test).

The *prePIP1* gene is abundantly expressed in *A. thaliana* roots. We therefore measured PIP-induced immunity in roots using a *MYB51p::GUS* reporter which was previously employed to monitor flg22-triggerred immune responses [36]. PIP1, PIP2, PEP1 and flg22 strongly activated *MYB51* promoter activity in the root elongation zone (EZ) (Figure 6A). MYB51-dependent indole-glucosinolate synthesis is required for callose deposition [37]. All peptides induced callose deposition in the root EZ (Figure 6A), while no such induction was detectable in the presence of CEP1 or IDL2 (Figure S7).

Given that *prePIP1* expression was induced upon *Foc* 699 infection, resistance against this pathogen was compared between WT and *35S::prePIP1* or *35S::prePIP2* plants. When *A. thaliana* seedlings were challenged with microconidia of *GFP*-labeled *Foc* 699 (*Foc* 699-*GFP*), fungal hyphae penetrated the EZ cortex 3–6 h post infection and reached the vascular tissue ∼12 hours later (Figure S8). However, the extent of *Foc* 699 penetration in the roots of *35S::prePIP1* and *35S::prePIP2* plants was significantly lower than in the roots of WT, as estimated from the GFP fluorescence signal (Figure 6B and C). When *Foc* 699 infected seedlings were potted into soil and left to grow for three weeks, the overexpression lines displayed a significantly reduced mortality compared to the WT plants (Figure 6D). These results indicate that overexpression of *prePIP1* or *prePIP2* enhances *Arabidopsis* resistance against *Foc* 699.

### RLK7 is the PIP1 receptor

**Figure 7:**
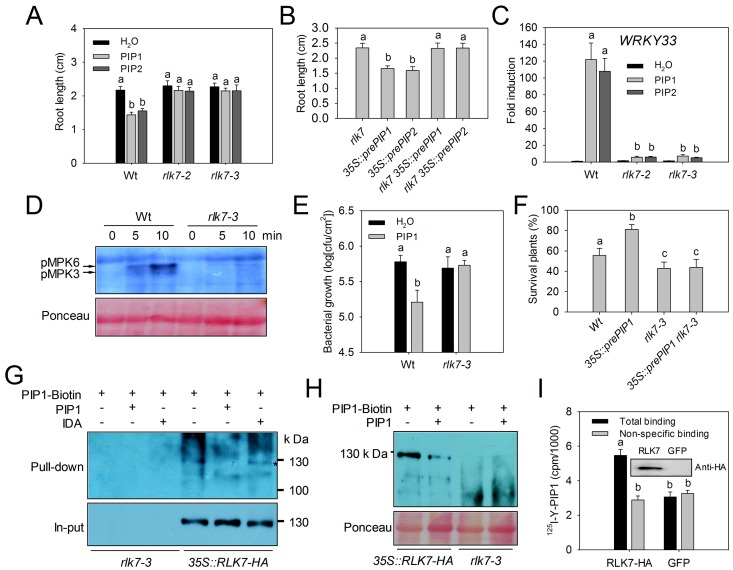
RLK7 is required for the PIP1 and PIP2 response and for PIP1 binding. (A) Root length of WT and *rlk7* seedlings grown with or without 1 µM PIP1 or 1 µM PIP2. (B) Root length of *rlk7* and *rlk7*×*35S::prePIP* seedlings. (A–B) Error bars represent the SE of the mean (n>30). Means marked by “a” differed significantly (p<0.001) from those marked “b” (*t*-test). (C) Transcription of *WRKY33* in WT and *rlk7* seedlings exposed to 1 µM PIP1 or 1 µM PIP2. Error bars represent the SE of the three replicates. Means marked by “a” differed significantly (p<0.001) from those marked “b” (*t*-test). (D) MAPK activation by PIP1 in WT and *rlk7-3* seedlings. Ten day old seedlings were exposed to 1 µM peptide for 5 and 10 min. Western blot analysis was performed with the phospho-p44/42 MAPK antibody. Two replicates were performed with similar results. (E) Growth of *Pst* DC3000 in WT and *rlk7-3* plants with or without treatment with 1 µM PIP1. Error bars represent the SE of the mean (n = 6). Three replicates were performed with similar results. Means marked by “a” differed significantly (p<0.01) from those marked “b” (*t*-test). (F) Survival rate of plants 21 days post infection with *Foc* 699-*GFP*. Error bars represent SE from three replicates that contained 30 to 40 plants each. Statistically significant (p<0.05) differences are indicated by different letters (*t*-test). (G) Detection of biotinylated PIP1 binding to RLK7-HA using a pull-down assay. Membrane proteins extracted from *rlk7* or *rlk7*/*35S::RLK7-HA* leaves incubated with PIP1-biotin bound to streptavidin beads in the presence (+) or absence (−) of unlabeled PIP1 or IDA. RLK7-HA bound to the beads was detected with an anti-HA antibody. (H) Detection of RLK7-HA by chemical cross-linking of PIP1-biotin. Cross-linking of PIP1-biotin to proteins from *35S::RLK7-HA* and *rlk7-3* plants in the presence (+) or absence (−) of excess unlabeled PIP1. Bands were detected with anti-biotin antibody. (I) ^125^I-Y-PIP1 binding activity of plasma membrane fragments from tobacco leaves expressing RLK7-HA or GFP. Error bars represent the SE of the mean (n = 5). Means marked by “a” differed significantly (p<0.01) from those marked “b” (*t*-test). (G–I) At least two repeats were performed with similar results.

Secreted peptides are typically recognized by plasma-localized LRR-RLKs [21]. The sequence similarity between PIPs and other SGP-rich peptides suggested that the hypothetical PIP1 receptor(s) could be structurally related to the CLV3p receptor CLV1 [38], the IDAp receptors HAE and HSL2 [35], or the PEP1 receptors PEPR1/2 [15], [16], all of which are class XI LRR-RLKs [5]. Like PEPR1/2 and other immune-related receptors, the hypothetical PIP1 receptor(s) is likely to be up-regulated by pathogen attack or PAMP induction. The *A. thaliana* genome harbors 28 category XI *LRR-RLKs* genes, six of which are induced by PAMP treatment or pathogen infection [27], [ 39]: *PEPR1/2*, *HAE*, *RLK7* (*At1g09970*), *At5g25930* (here named *HSL3*), and *SOBIR1*. The *SOBIR1* product was shown to act as a co-regulator of multiple receptor-like proteins (RLPs) that are involved in immune recognition [40]–[42], and was suggested not to function directly in ligand recognition due to its short LRR domain. To identify the putative receptors of PIP1 and PIP2, we analyzed the response of T-DNA insertion mutants of *RLK7*, *HAE*, *HSL2*, *HSL3*, and *FLS2* to PIP1 and PIP2 treatments. No inhibition of root growth was observed in two *rlk7* mutants, *rlk7-2* and *rlk7-3*, while the other mutants responded similar as the WT (Figures 7A, S9). The roots of *35S::prePIP1* or *35S::prePIP2* plants were significantly shorter than those of WT plants, while roots of the double homozygous F2 progeny of a cross between *35S::prePIP1* or *35S::prePIP2* and *rlk7-3* grew normally as did those of *rlk7* mutants. Thus, inhibition of root growth by prePIP1 and prePIP2 is RLK7 dependent (Figure 7B).

In contrast to the WT, the *rlk7-3* plants failed to up-regulate expression of *FRK1*, *WRKY33*, and *WRKY53* upon treatment with PIP1 or PIP2 (Figure 7C and S10A and B). In contrast, flg22 strongly induced expression of *FRK1* both in WT and *rlk7-3* plants, but not in the *fls2* mutant (Figure S10C), suggesting that RLK7 responds specifically to PIPs. Moreover, PIP1-induced MPK3 and MPK6 phosphorylation was also abolished in *rlk7-3* (Figure 7D), as was the increase of host resistance against *Pst* DC3000 infection by pre-treatment of *Arabidopsis* leaves with PIP1 (Figure 7E). The *prePIP1* overexpression line displayed a significantly reduced mortality compared to the WT plants as indicated above, while the double homozygous F2 progeny of a cross between *35S::prePIP1* and *rlk7-3* displayed a higher mortality as did those of *rlk7-3* mutants (Figure 7F).

We next asked whether RLK7 directly binds the PIP1 peptide. This was first addressed through a pull-down assay with biotinylated PIP1 in *A. thaliana* plants expressing hemagglutinin (HA) tagged-RLK7 (RLK7-HA). Two derivatives of biotin labeled PIP1 (Biotin-PIP1 and PIP1-biotin) were confirmed to maintain their biological function by determining their activities on root growth inhibition and marker gene induction (Figure S11). Since PIP1-biotin exhibited a higher activity, it was used for all subsequent experiments. We found that RLK7-HA was pulled down with PIP1-biotin-associated streptavidin beads from membrane protein extracts of *rlk7-3* plants harboring RLK7-HA, but not from *rlk7-3* plants (Figure 7G). Binding of RLK7-HA to the beads was inhibited by a 100× excess of unlabelled PIP1 but not by unlabelled IDA. Next, a chemical cross-linking assay was employed to prove a direct binding of PIP1-biotin to RLK7-HA. PIP1-biotin peptide was incubated with protein extracts of *RLK7-HA* transgenic plants or *rlk7-3* mutants, and cross-linked with its potential receptor using a chemical cross-linker. After separation by SDS-PAGE, protein samples were hybridized with an anti-biotin antibody. A protein of 130 kD, consistent with the molecular mass of RLK7-HA, was detected in *RLK7-HA* plants but not in *rlk7-3* mutants (Figure 7H), suggesting that the protein corresponds to the RLK7-HA protein. Binding of PIP1 to RLK7 was further corroborated using a photoaffinity labeling assay. *RLK7*-*HA* or *GFP* (negative control) were transiently expressed in tobacco leaves, and homogenized leaf tissues were incubated with 1 nM ^125^I-labeled PIP1 in the presence or absence of 10 µM unlabeled PIP1. Specific binding of ^125^I-labeled PIP1 was detected in the homogenate from leaves expressing RLK7-HA protein, but not in those from leaves expressing GFP (Figure 7I).

### PIP1-RLK7 signaling is partially dependent on BAK1, but independent of BIK1

**Figure 8:**
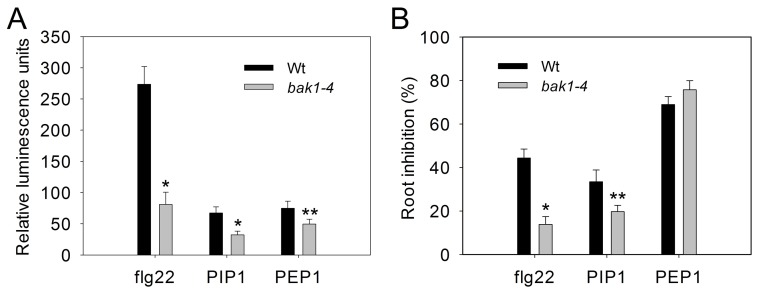
Full PIP1 response requires *BAK1*. (A) PIP1-induced ROS production in *bak1-4* leaves. ROS production was measured after elicitation with 1 µM peptides. Error bars represent the SE of the mean (n = 5). (B) PIP1-induced root growth inhibition. Error bars represent the SE of the mean (n>30). *, **: significantly different from mock treatment at p<0.001 and <0.01 (*t*-test). Three repeats were performed with similar results.

The receptor kinase BAK1 plays an important role in PTI immune activation by forming heteromeric co-receptor complexes with multiple LRR-RLK receptors, including FLS2 and PEPR1 [7], [8], [39]. Sensitivity to flg22 and PEP1 was partially reduced in *bak1* T-DNA insertion mutants *bak1-3* and *bak1-4* [43]. While dimerization of FLS2 with BAK1 occurs after flg22 perception by FLS2, PEPR1 interacts constitutively with the kinase domain of BAK1. Since PIP1 triggers similar early immune responses as flg22 and PEP1, we asked whether BAK1 also contributes to PIP1 responses. Indeed, PIP1-induced ROS production and root growth inhibition were both reduced in *bak1-4* than in WT plants (Figure 8A and B). In contrast, while PEP1-induced ROS production was also reduced in the *bak1-4* mutant, inhibition of root growth was unaffected (Figure 8A and B). Thus, while PIP1-RLK7 signaling is partially dependent on BAK1, PIP1 and PEP1-induced responses have different requirements for BAK1.

**Figure 9:**
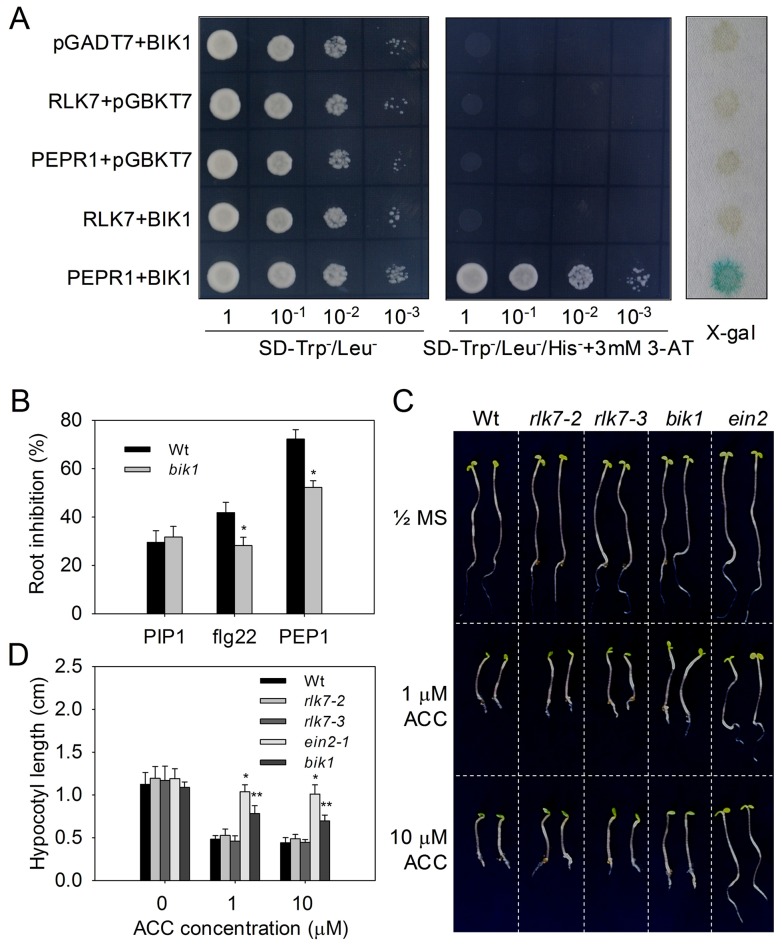
PIP1-RLK7 signaling is *BIK1* independent. (A) Interaction between PEPR1 or the RLK7 kinase domain and BIK1 in the yeast two-hybrid assay. Yeast cells containing the indicated plasmids were analyzed for *His* and *LacZ* reporter activities. PEPR1_KD_, pGADT7 containing *PEPR1* kinase domain; RLK7_KD_, *pGADT7* containing *RLK7* kinase domain; BIK1, pGBKT7 containing *BIK1*. (B) Root length of 8-day old WT and *bik1* seedlings grown in the presence of 1 µM flg22, PIP1, or PEP1. Triple response phenotype (C) and hypocotyl length (D) of *A. thaliana* seedlings grown in the presence or absence of ACC. Error bars represent the SE of the mean (n>30). *, **: significantly different from mock treatment at p<0.001 and <0.01 (*t*-test). At least two repeats were performed for all experiments with similar results.

FLS2 and PEPR1 initiate downstream signaling by directly interacting with the receptor-like cytoplasmic kinase BIK1 [9], [17]. Therefore, we investigated the possible interaction between BIK1 and RLK7. Yeast two-hybrid results did not indicate an interaction between BIK1 and the kinase domain of RLK7, while confirming the interaction between BIK1 and the kinase domain of PEPR1 reported previously (Figure 9A). In plants lacking *BIK1*, flg22- and PEP1-induced root growth inhibition was attenuated while the effect of PIP1 was unchanged (Figure 9B). Given the known role of PEPR1-BIK1 in mediating ET responses [17], we compared hypocotyl elongation in WT and *rlk7* seedlings treated with ACC, but found no significant difference (Figure 9C and D). However, sensitivity to ACC treatment was attenuated in both *ein2* (*ethylene insensitive 2*) and *bik1* mutants. Taken together, these results suggest that PIP1-RLK7 signaling is independent of BIK1.

### PIP1-RLK7 and PEP1-PEPR1 cooperate to amplify FLS2 signaling

**Figure 10:**
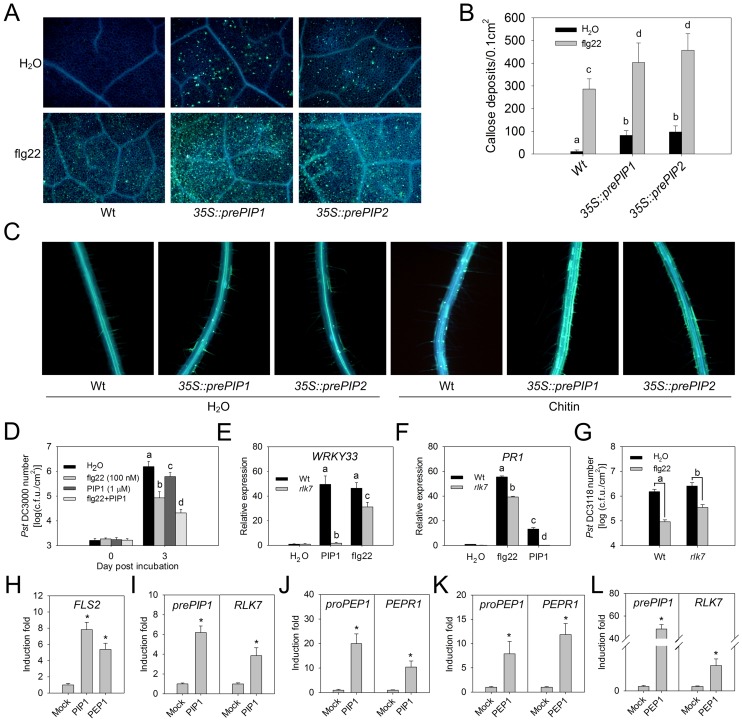
PIP1-RLK7 and PEP1-PEPR1 cooperatively amplify FLS2 signaling. (A) Fluorescence microscopy imaging and (B) quantification of flg22-induced callose deposition in leaves of WT and *prePIP* over-expression lines. Error bars represent the SE of the mean (n>10). Statistically significant (p<0.01) differences indicated by different letters (*t*-test). Two repeats were performed with similar results. (C) Fluorescence microscopy imaging of chitin-induced callose deposition in roots of WT and *prePIP* over-expression lines. Two repeats were performed with similar results. (D) *Pst* DC3000 growth in *A. thaliana* leaves pretreated with flg22, PIP1 or a combination of flg22 and PIP1. Error bars represent the SE of the mean (n = 8). Three repeats were performed with similar results. (E) RT-qPCR analysis of *WRKY33* transcript abundance after 30 min treatment with H_2_O or 1 µM peptide. Error bars represent the SE of the three repeats. (F) RT-qPCR analysis of *PR1* transcript abundance after 24 h treatment with H_2_O or 1 µM peptide. Error bars represent the SE of the mean (n = 3). Two repeats were performed with similar results. (D–F) Statistically significant (p<0.01) differences were indicated by different letters (*t*-test). (G) *Pst* DC3118 growth in leaves of WT and *rlk7* plants treated with water (mock) or 100 nM flg22. Error bars represent the SE of the mean (n = 8). Statistically significant (p<0.01) differences were indicated by different letters (ANOVA). Three repeats were performed with similar results. (H) Fold induction of *FLS2* expression by treatment with PIP1 and PEP1. (I) Fold induction of *prePIP1* and *RLK7* by PIP1. (J) Fold induction of *proPEP1* and *PEPR1* by PIP1. (K) Fold induction of *proPEP1* and *PEPR1* by PEP1. (L) Fold induction of *prePIP1* and *RLK7* by PEP1. (H–L) *A. thaliana* seedlings were treated with 1 µM PIP1 or PEP1 for 0.5 hours, and gene expression was measured by RT-qPCR analysis. Error bars represent the SE of the mean (n = 3). *: significantly different from mock treatment at p<0.01 (*t*-test). Two repeats were performed with similar results.

Because the expression of *prePIP1* and *RLK7* is induced by flg22 and PIP1 triggers a similar immune response to flg22, we hypothesized that PIP1-RLK7, like PEP1-PEPR1, may serve to amplify PAMP signaling. In support of this idea, flg22- or chitin-induced callose deposition was more pronounced in leaves and roots of *35S::prePIP1* or *35S::prePIP2* plants than in WT plants (Figure 10A–C). Moreover, we observed an additive effect in elevation of host resistance against *Pst* DC3000 in plants pre-treated simultaneously with flg22 and PIP1, compared to each single peptide elicitor (Figure 10D). Furthermore, activation of *WRKY33* and *PR1*, two genes representing, respectively, early- and late-response immune reporters, by flg22 was reduced in *rlk7* plants compared to WT plants (Figure 10E and F), and the level of flg22-induced host resistance against *Pst* DC3118 (a coronatine deficient *Pst* DC3000 mutant) was less marked in the *rlk7* mutant (Figure 10G). Finally, PIP1 and PEP1 both appeared to enhance flg22 responses via up-regulation of *FLS2* expression (Figure 10H).

A crosstalk between PIP1 and PEP1 signaling was further supported by the finding that PEP1-induced root growth inhibition and *WRKY33* expression were impaired in mutants lacking *RLK7* (Figure S12). Either PEP1 or PIP1 induced the transcription of all the genes encoding precursors and receptors of the two peptides (Figure 10I–L). Thus, PIP1-RLK7 and PEP1-PEPR1 act cooperatively to amplify FLS2-initiated immunity.

## Discussion

### PIP1 is a functional secreted peptide

The identification of elicitors to date has relied on various bioassays conducted on extracts of pathogen and/or host tissue [3], [44], [45]. Because the active components are typically present in low abundance, this mode of analysis is technically challenging. With the widespread development of genomic and transcriptomic data in *A. thaliana*, bioinformatics is increasingly offering potential for predicting the identity of elicitors. Here, by analyzing PAMP-induced gene transcription data, a gene family encoding precursors of the secreted peptide elicitors PIPs was identified.

The release from precursor proteins by proteolysis in the extracellular space is a critical process for secreted peptides [20]. *In vitro*, prePIPs are typically cleaved close to the C terminus. Specific cleavage was confirmed *in vivo*, since recombinant GST-ΔP1 protein suffered a similar processing pattern when injected into leaves of *A. thaliana*. Although it is generally assumed that mature peptides are released from precursors through endopeptidase-mediated cleavage [46], the only cleavage recognition site identified so far in *A. thaliana* is a specific sequence in the peptide PSK4 which was confirmed to be proteolytically cleaved by the subtilase SBT1.1 [46]. In most post-translationally modified secreted peptide precursors, cleavage occurs before or after Arg, Asp, His or Asn residues located at both sites of the C-terminal conserved motifs [20]. Members of the prePIP1 family harbor a conserved Arg or His residue at each side of the SGPS-motif. We found that exogenous application of synthetic PIP1 peptide corresponding to the conserved SGPS-motif successfully mimicked the phenotypes of *A. thaliana* plants transiently or constitutively expressing *prePIP1*. This indicates that PIP1 is a biologically active form derived from prePIP1, and shares part or all of the sequence with the genuine mature peptide cleaved from the precursor. However, considering that PIP1 peptide was saturated at micromolar concentration in root growth inhibition assays, we cannot exclude the presence of a more active peptide. Further, a mass spectrometry analysis is needed to confirm the cleavage site and to identify the mature peptides cleaved from prePIPs precursors.

Proline hydroxylation is common in SGP-rich peptides such as CLV3p and CEP1 [33], [34]. PIP family members harbor two conserved proline residues. A comparison of the root growth inhibitory effect of proline hydroxylated and non-hydroxylated forms of PIP1 revealed that hydroxylation enhances the biological activity of the peptide. In contrast, unmodified CLV3 and hydroxylated CLV3 peptides had similar activities in root growth inhibition [32], [33]. This suggests that proline hydroxylation differentially affects the biological activities of PIP1 and CLV3. It is currently not clear whether proline hydroxylation of PIP1 affects its affinity for the receptor or its stability.

### PIP1 activates plant immunity in an RLK7-dependent manner

We found that the PIP1 and PIP2 peptides activate similar immune responses as flg22 and PEP1, including expression of marker genes, ROS production, callose deposition and MAPK activation. The possibility that this result was caused by contamination with flg22 and/or PEP1 can be excluded for several reasons. First, independently synthesized PIP peptides exhibited the same activity; second, IDL2p and CEP1, two peptides with a similar sequence structure to PIPs that were synthesized together with PIPs, failed to activate immune responses; third, a *fls2* loss-of-function mutant that is insensitive to flg22 still responded to PIPs; and fourth, PIP1 and PEP1 differed functionally from each another.

A reverse genetics screen identified the class XI LRR-RLK RLK7 as the responsible for PIP1- and PIP2-triggered responses. RLK7-PIP1 binding data implicate that RLK7 acts as the PIP1 receptor. However, the flg22 receptor FLS2 which was previously proposed to perceive CLV3p and Ax21 [23], [47], [48], failed to recognize PIP1 since *fls2* mutants were still responsive to PIP1-induced up-regulation of *FRK1*. Although RLK7 was required for the PIP1-induced enhancement of host resistance against *Pst* DC3000, loss-of-function *rlk7* mutants showed no reduction in the level of resistance in the absence of PIP1 treatment. This is reminiscent of the finding that the *pepr1*/*pepr2* double mutant is not affected in the level of resistance against *Pst* DC3000 [16]. The virulence of *Pst* DC3000 relies heavily on secreted effector proteins which can suppress host immunity by blocking various signaling pathways [49]. The resistance conferred by the PIP-RLK7 signaling pathway may thus be severely disrupted by pathogen effectors. Moreover, the expression pattern of *prePIP1* suggests that PIP1-RLK7 resistance is perhaps more specific to pathogens infecting through the hydathodes or proliferating in the vascular tissue. This idea is consistent with the high host resistance conferred by *prePIP1* or *prePIP2* over-expression against the fungus *Foc* 699, a soil-borne pathogen that colonizes the root vascular tissue.

### PIP1-RLK7 share overlapping but also distinct signaling components with PEP1-PEPR1

PIP1 activates an almost identical set of signaling events as flg22 and PEP1, suggesting that the three pathways likely share a number of components. BAK1 regulates several of the immune signaling pathways triggered by LRR-RLK type immune receptors, including FLS2 and PEPR1 [7], [8], [39]. We found that PIP1-RLK7 mediated responses are less pronounced in *bak1-4* mutants, suggesting that BAK1 contributes to PIP1-RLK7 signaling. Previous studies suggested that BAK1 and BAK1-LIKE1 (BKK1) function in parallel in FLS2- and PEPR1-activated immune signaling, since the *bak1* mutant is only partially insensitive to flg22 and PEP1 while the *bak1*/*bkk1* double mutant is completely insensitive [7], [39], [43]. We noted that the *bak1-4* mutant retained some sensitivity to PIP1, implying some degree of redundancy between BAK1 and BKK1. However, both flg22- and PIP1-induced ROS production and root growth inhibition were attenuated in the *bak1-4* mutant, whereas only ROS production was affected upon induction with PEP1. This suggests possible differences in the requirement for BAK1 between flg22, PEP1 and PIP1 responses.

BIK1, another important regulator of the FLS2 and PEPR1 signaling pathways, is rapidly phosphorylated when flagellin binds to FLS2 [9]. BIK1 phosphorylation can also be induced with PEPR1 or PEPR2 in the presence of ET or PEP1 [17]. This is consistent with the results from our root growth inhibition assay and previous ET-induced triple response analysis. No direct protein-protein interaction between RLK7 and BIK1 could be detected by yeast two-hybrid analysis, and no parallels were found between PIP1-RLK7 and PEP-PEPR1 in the context of the ET response. Neither did the *rlk7* mutants show reduced sensitivity to ACC, nor was PIP1-induced root growth inhibition attenuated in the *bik1* mutant. BIK1 is a member of class VII RLCKs, which have been suggested to integrate immune signaling in *A. thaliana* from cell-surface-localized receptors [9], [10]. Thus it is possible that other members of class VII RLCKs mediate RLK7 signaling and are responsible for the observed differences in signaling outputs between RLK7 and PEPR1.

ProPEP1 family members lack a classical signal peptide, and therefore the mechanism underlying PEP release is unclear. Since expression of *proPEP1* is up-regulated by wounding and treatment with the wound signal MeJA, it was suggested that release of PEP1 from plant cells may be the result of cell injury caused by pathogen attack or wounding [3]. Consistent with this, PEPR signaling was recently shown to operate predominantly at local pathogen challenged sites, though systemic immunity can be activated by treatment with PEP1 [50]. In contrast, PIP1 is secreted into the extracellular spaces through a cell-autonomous secretory pathway and massive expression of *prePIP1* is detected in vascular tissues, suggesting that PIP1 is likely to act as a mobile signal involved in systemic immune activation.

### PIP1-RLK7 and PEP1-PEPR1 cooperatively amplify FLS2 signaling

**Figure 11:**
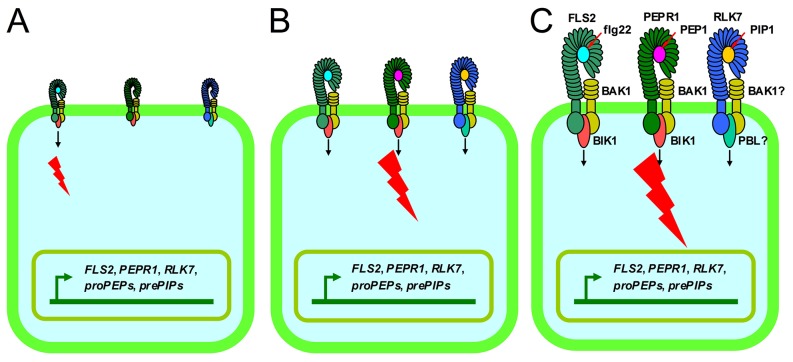
Proposed model of the roles of PIP1-RLK7 and PEP1-PEPR1 in PTI signal amplification. (A) flg22 perception by FLS2 primes immunity and activates transcription of *FLS2*, *PEPR1*, *RLK7*, *proPEP1* and *prePIP1*. (B) PEP1 and PIP1 peptides are generated from their precursor proteins and released into the apoplast to trigger PTI responses after recognition by the cognate receptors. Moreover, they act in a positive feedback loop by activating expression of genes encoding their own precursors and receptors, as well as FLS2. (C) Finally, the level of immunity is maximized by the combined effect of FLS2, PEPR1 and RLK7.

Activation of immunity by endogenous signals is a common strategy exploited by animals and plants to amplify immune responses after perceiving a limited number of invading pathogens [51]. In animals, many endogenous peptides such as interleukins which are generated upon PAMP recognition, were confirmed to function in inflammation [52]. In plants, PEP1 was suggested to act as a PTI amplifier because (1) PAMP treatment increases transcription of *proPEP1*, (2) PEP1 and PAMPs activate similar immune responses, and (3) PEP1 receptors are required for full activation of PTI signaling and resistance against bacterial infection [16], [18], [53]. In this study, *prePIP1* and *RLK7* were induced by flg22, and flg22-triggered immunity was impaired in *rlk7* mutants. These findings imply that PIP1-RLK7 and PEP1-PEPR1 have similar functions in FLS2 signal amplification. PIP1 and PEP1, respectively, induce their corresponding precursor and receptor genes showing that self-amplification mechanisms act in both signaling pathways. Importantly, PIP1 and PEP1 also induce the expression of each other's precursor and receptor genes. Further, the level of PEP1 responses was decreased in *rlk7* mutants. These demonstrate that the two endogenous peptide signaling pathways are interdependent and cooperate to amplify the immune response. We propose a working model (Figure 11) in which FLS2 signaling is initially primed by the perception of flg22, followed by upregulation of the host peptide elicitors PIP1 and PEP1 and their respective receptors PEPR1 and RLK7. Once PIP1 and PEP1 are released and processed in the apoplast, they initiate the immune response and also increase expression of *prePIP1*, *RLK7*, *proPEP1*, *PEPR1* and *FLS2*, leading to an amplification of the immune responses via the combined effect of FLS2, PEPR1 and RLK7.

## Materials and Methods

### Plant materials

*A. thaliana* were grown in potting mix or on 1/2 MS medium (containing 1/2 MS salts, 1% w/v sucrose and 0.8% w/v agar, pH 5.7) in a controlled growth chamber providing a 10 h photoperiod (140 µmol•m^−2^•s^−1^ light) at 22°C/20°C day/night and 60% relative humidity. *fls2* [54], *rlk7* [55], *hae*/*hsl2* [35], *ein2-1* [17], *bak1-4* [7], and *bik1* [10] mutants used were described earlier. Verification of homozygous T-DNA insertion mutants was carried out by a PCR assay based on locus-specific primers (Table S3).

### Root growth inhibition assay

Arabidopsis seedlings were germinated on 1/2 MS media, and then transferred to 1/2 MS liquid medium (1/2 MS salts, 1% sucrose, pH 5.7) adding various concentrations of PIP1 or other peptides in a 6-well plate. The length of the seedling roots was measured after 5–7 days.

### Constructs

*PrePIP1*, *prePIP2*, and *RLK7* coding sequences were PCR-amplified from *A. thaliana* genomic DNA using locus-specific primers, and the products were separately inserted into *pCAMBIA1300-HA* vector downstream of the CaMV 35S promoter to generate *pCAMBIA1300-35S::prePIP1-HA*, *pCAMBIA1300-35S::prePIP2-HA* and *pCAMBIA1300-35S::RLK7-HA*. An ∼2.8 kb fragment upstream of the *prePIP1* start codon was amplified from *A. thaliana* genomic DNA and inserted into the *pGFPGUSPlus* vector [56] to construct *prePIP1p::GUS* and *prePIP1p::GFP*. Truncated *prePIP1*, *prePIP2* and *prePIPL5* coding sequences were amplified from *A. thaliana* genomic DNA using locus-specific primers and inserted into *pGEX-6p-1* to generate *GST-ΔprePIP1*, *GST-ΔprePIP2*, *GST-ΔprePIPL5*. The *BIK1* coding sequence was amplified from *A. thaliana* cDNA and inserted into *pGBKT7* to generate *pGADT7-BIK1*. The sequences encoding the kinase domains of *PEPR1* (residues 827–1123) and *RLK7* (residues 671–977) were amplified from *A. thaliana* cDNA and inserted into *pGADT7* to generate *pGADT7-PEPR1_KD_* and *pGADT7-RLK7_KD_*. All the sequences primers are listed in Table S3.

### Synthetic peptides

Peptides of purity level 98% were synthesized by Yaguang Biochemical Company (Shanghai, China). Their sequences are given in Table S2.

### Transient expression in tobacco leaves

Transient expression in tobacco leaves was performed as described previously [57]. *Agrobacterium tumefaciens* strain GV3101 harboring *pCAMBIA1300-RLK7-HA*, *pCAMBIA1300-GFP*, *pCAMBIA1300-prePIP1-GFP* or *pCAMBIA1300-CLV3-GFP* were grown overnight in YEB medium and transferred to 1/2 MS liquid medium containing 50 µM acetosyringone for 4 h until an OD_600_ of 0.4–0.6 had been reached. The culture was then diluted 1∶1 with 10 mM MES (pH 5.6), 10 mM MgCl_2_, 150 µM acetosyringone, and pressure-infiltrated into the leaves of 4–5 week old tobacco plants. Transfected leaves were collected after 48–72 h.

### Proteolytic processing assays

*In-vitro* cleavage assays were performed as described previously [58]. In brief, *GST*-tagged truncated *prePIPs* (*GST-ΔPIPs*) were expressed in *E. coli* BL21 (DE3) and purified using glutathione Sepharose (GE Healthcare). The purified proteins were incubated with *Arabidopsis* protein extracts or BSA (control) for 0–2 h at room temperature. The samples were then subjected to SDS-PAGE to determine the protein composition. For the *in-vivo* cleavage assay, GST-PIP1 (1 µg/µL) or GST (1 µg/µL) was syringe-injected into *A. thaliana* leaves and incubated for 2 h, then extracellular fluids were extracted and analyzed by SDS-PAGE.

### GUS staining

GUS staining was performed as described previously [36]. In brief, plant tissues were immersed in staining buffer (100 mM sodium phosphate buffer, pH 7.0, 10 mM EDTA, 1 mM potassium ferrocyanide, 1 mM potassium ferricyanide, 1 mM X-Gluc, and 0.1% Triton X-100) and incubated at 37°C for 2–6 h. Stained samples were cleared in 70% ethanol and observed by the Olympus BX53 microscope.

### Luciferase reporter assay

Protoplast transfection and subsequent luciferase reporter assay were performed as described previously [14]. *FRK1p-LUC* reporter was co-transfected with *prePIP1* constructs and *UBQ10p-GUS* (internal control). After 6 hours' incubation, luciferase activities were tested with a Luciferase Assay kit and a GloMax-20/20 luminometer (Promega). For analysis of *FRK1p-LUC* induction by exogenous application of peptide elicitors, protoplasts were incubated overnight after transfection with *FRK1p-LUC* reporter, and then were induced with 1 µM peptide for 4 hours before detection of luciferase activity.

### Quantitative RT-PCR analysis

Total RNA was extracted from plant tissues by the TRIzol reagent (Invitrogen) following the manufacturer's protocol. A 2 µL aliquot of the total RNA preparation was subjected to reverse transcription using a RevertAi First Strand cDNA Synthesis kit (Fermentas). The resulting cDNA was amplified using the SYBR Green Mix (Roche) and gene-specific primers (Table S3). *AtActin2* was used as the reference sequence.

### ROS measurement

A luminol-based assay was used to quantify ROS in treated leaves [59]. The same amount of 1–2 mm leaf fragments cut from Arabidopsis leaves were incubated in 100 µL water for 12 h, and then 100 µM luminol (Sigma), 10 µg/mL horseradish peroxidase (Sigma) and 1 µM peptide were added rapidly in turn. The resulting luminescence was measured using a GloMax-20/20 luminometer (Promega) at one minute intervals over 15 min.

### Aniline blue staining

Staining of callose deposits was achieved following methods described previously [36], [59]. Adult leaves were infiltrated with either water or 1 µM peptide for 8 h, and the roots of 10-day old seedlings were immersed in 1/2MS liquid medium with or without peptides (1 µM) or chitin (500 µg/L) for 18 h. The materials were then fixed in 3∶1 ethanol∶acetic acid for 6 h, changing the fixative solution every 2 h. The samples were rehydrated in 50% ethanol for 2 h, and then thoroughly rinsed in water. Finally the samples were incubated in staining solution (150 mM K_2_HPO_4_ (pH 9.5), 0.01% (w/v) aniline blue, Sigma-Aldrich) for 30 min. Callose was visualized using UV-epifluorescence microscopy. Signal intensities were estimated using Image J software.

### MAPK assay

Ten seedlings were immersed in sterile water overnight. Peptides were then added to a final concentration of 1 µM for 5–15 minutes induction. After induction, the seedlings were snap-frozen in liquid nitrogen and ground to a fine powder, from which total protein was extracted by suspension in 50 mM HEPES (pH 6.8), 150 mM NaCl, 1% (w/v) SDS, 2 mM DTT, 10 mM NaF, 10 mM NaVO_3_, 5 mM EDTA, 1× protease inhibitor cocktail (Roche). An anti-phospho p44/p42 MAPK antibody (Cell Signaling Technology) was used to detect active MPK6 and MPK3 via immunoblotting.

### Binding assay

Y-PIP1 peptide was labeled with ^125^I as described previously [60]. In brief, 2 nmol Y-PIP1 peptide and 600 µCi Na^125^I (PerkinElmer) dissolved in 100 µL sodium phosphate buffer (10 mM, pH 7.4) were added into a glass vial pre-coated with 1,3,4,6-tetrachloro-3α,6α-diphenylglycouril, and were incubated for 15 min at root temperature. After passing through a Sephadex G25 column (PD-10 column, GE Healthcare), ∼800 µL ^125^I-Y-PIP1 containing 1.7×10^7^ counts per minute (cpm) was collected. Plasma membrane fragments were extracted from 200 mg tobacco leaves and re-suspended in binding buffer (25 mM MES, pH 6.0, 3 mM MgCl_2_, 10 mM NaCl, 2 mM dithiothreitol and protease inhibitor cocktail (Roche)) with a final concentration of 2 µg/µL total protein. The plasma membrane (100 µL) was incubated with 2 µL ^125^I-Y-PIP1 (∼100 fmol) in the presence or absence of 10 µM unlabelled PIP1 for 15 min at 4°C, then were collected by a vacuum filtration system through glass fibre filters (Millipore, 2.5-cm diameter). After washed with cold washing buffer (binding buffer supplemented with 1% BSA, 1% bactotrypton, 1% bactopepton), the binding was determined by γ-counting.

### Biotinylated-PIP1 pull-down assay

Plasma membrane proteins were extracted from the Arabidopsis leaves of *rlk7* mutant and *rlk7*/*35S::RLK7-HA* with an extraction buffer (25 mM MES/KOH (pH 6.0), 3 mM MgCl_2_, 10 mM NaCl, 0.5% SDS and 1× protein inhibitor cocktail (Roche)), then were diluted ten folds with a binding buffer (25 mM MES/KOH (pH 6.0), 3 mM MgCl_2_, 10 mM NaCl and 1× protein inhibitor cocktail (Roche)). Biotinylated PIP1 (1 µg) was coupled to 20 µL streptavidin beads (Pierce) for 1 h at 4°C. After three rinses in 500 µL binding buffer, the beads were incubated with 200 µL of the prepared plasma membrane proteins in the presence or absence of 100× excess of unlabelled PIP1 or IDA for 2 h at 4°C. After rinsed three times in 500 µL binding buffer, the beads were boiled for 5 minutes in 50 µL 1× Laemilli buffer. The RLK7-HA was detected with an anti-HA monoclonal antibody (Qiagen).

### Chemical cross-linking

Chemical cross-linking of PIP1-biotin to RLK7 was displayed as described previously. PIP1-biotin (1 µM) was incubated with the total protein (50 µg) extracted from *rlk7* or *355S::RLK7-HA* plants in the presence or absence of excess (50 µM) unlabeled PIP1 for 30 min at 4°C. After adding 1/10 volume of 25 mM EGS (Pierce), the mixture was incubated for another 30 minutes at room temperature before the reaction was terminated by the addition of 1 µL Tris-HCl buffer (1 M, pH 7.5). Proteins in samples were separated by SDS-PAGE and detected with anti-biotin antibody (Cell Signaling Technology).

### Yeast two-hybrid assay

Interactions between BIK1 and the kinase domain of PEPR1 (residues 827–1123) or RLK7 (671–977) were tested using the GAL4 yeast two-hybrid system (Clontech). In brief, the *pGADT7-PEPR1_KD_* or *pGADT7-RLK7_KD_* plasmid was co-transfected with *pGBKT7-BIK1* into *Saccharomyces cerevisiae* strain AH109. The transformed yeast cells were spotted on a synthetic dropout (SD) medium (Difco Yeast Nitrogen Base) lacking tryptophan, leucine, and histidine (SD-Y^−^-L^−^-H^−^) but supplementing with 3 mM 3-amino-1,2,4-triazole (3-AT, Sigma) to detect the *His* reporter activity. Transformants were also detected on the basis of *lacZ* reporter activity with 50 µg/mL X-gal dissolved in 25 mM phosphate buffer.

### Pathogen inoculations and quantification

*Pst* DC3000 inoculation assay was performed as described previously [61]. The bacterial suspension (2×10^5^ colony-forming units (cfu)/mL) with or without 1 µM peptide was syringe infiltrated into leaves of 5-week old *A. thaliana* plants. *Foc* 699-*GFP* strain was obtained by cotransformation of the *F. oxysporum* f. sp. *conglutinans* strain 699 with the *sGFP* coding region driven the *Aspergillus nidulans gpdA* promoter and the trpC terminator, and the hygromycin resistance cassette, as described previously [62], [63]. *Foc* 699-*GFP* was grown in half strength potato dextrose broth at 28°C for 2 to 3 days. Ten day old seedlings were exposed to a 2 mL volume of a microconidia suspension (1×10^6^ spores/mL sterile water) and incubated for 3–24 h at 22°C. To quantify *Foc* 699-*GFP* biomass, genomic DNA was extracted from 30 infected seedlings after rinsing them three times in sterile water, and used as a template for qPCR with *GFP*-specific primers (Table S3). The *AtActin2* gene was used as the reference sequence. To monitor infection, Arabidopsis seedlings were rinsed three times with sterile water after 6-hour incubation with spore solution, planted into soil, and survival of the plants was assessed after 21 days.

### Accession numbers

Sequence information of genes involved in this article can be found in the Arabidopsis information resource or the Arabidopsis unannotated secreted peptide database under the following accession numbers: At4g28460 (*prePIP1*), At4g37290 (*prePIP2*), At2g23270 (*prePIP3*), At1g49800 (*prePIPL1*), At3g06090 (*prePIPL2*), At4g37295 (*prePIPL3*), At5g43066 (*prePIPL4*), ath_mu_ch1_43150top (*prePIPL5*), ath_mu_ch5_43674top (*prePIPL6*), ath_mu_ch4_17161top (*prePIPL7*), ath_mu_ch5_43661top (*prePIPL8*), At1g09970 (*RLK7*), At5g46330 (*FLS2*), At1g73080 (*PEPR1*), At1g17750 (*PEPR2*), At2g31880 (*SOBIR1*), At4g28490 (*HAESA*), At5g65710 (*HSL2*), At5g25930 (*HSL3*), At5g64900 (*proPEP1*), At4g33430 (*BAK1*), At2g39660 (*BIK1*), At5g24110 (*WRKY30*), At2g38470 (*WRKY33*), At4g23810 (*WRKY53*), At2g19190 (*FRK1*), At2g14610 (*PR1*), At5g44420 (*PDF1.2*), At1g18570 (*MYB51*), At5g03280 (*EIN2*), At1g68765 (*IDA*), At5g64667 (*IDL2*), At1g47485 (*CEP1*).

## Supporting Information

Figure S1 **SGPS-motif of** ***prePIP*** **homologs in various plants.** (A) Multiple sequence alignments of the conserved C-termini in prePIP homologs. (B) A neighbor-joining phylogenetic tree of the C-terminal sequences in prePIP homologs. GenBank accession numbers are as follows: ACU15907 (GmPIPL1), NP_001238364 (GmPIPL2), XP_006606893 (GmPIPL3), NP_001239759 (GmPIPL4), ACG48199 (ZmPIPL1), ACG26477 (ZmPIPL2), NP_001175941 (OsPIPL1), XP_003632092 (VvPIPL1), XP_003589124 (MtPIPL1), XP_003606833 (MtPIPL2), XP_002534518 (RcPIPL1), XP_002322914 (PtPIPL1), XP_002462659 (SbPIPL1).
(TIF)

Figure S2 ***A. thaliana*** **SGP-rich peptide sequences.** (A) Multiple sequence alignment. (B) A neighbor-joining phylogenetic tree.
(TIF)

Figure S3 **Expression and purification of GST and GST-ΔprePIPs (GST-ΔPs) from E.** ***coli*** **strain BL21 (DE3).** Proteins were separated by SDS-PAGE and detected using Coomassie Brilliant Blue staining. Arrows mark the expressed GST and GST-ΔPs.
(TIF)

Figure S4 **GST-ΔP1 cleavage** ***in vivo*** **. GST-ΔP1 or GST (control) was injected into** ***A. thaliana*** **leaves.** Extracellular fruit was extracted for SDS-PAGE detection. Dots mark intact GST-ΔP1, triangles processed GST-ΔP1. Two repeats were performed with similar results.
(TIF)

Figure S5 **The floral abscission region of** ***A. thaliana*** **over-expressing** ***prePIP1*** **,** ***prePIP2*** **,** ***IDA*** **and At5g05300 (bar = 1 mm).**
(TIF)

Figure S6 **Transcript abundance of** ***FRK1*** **,** ***WRKY33*** **,** ***WRKY53*** **, and** ***PR1*** **upon induction with flg22, PEP1, PIP1 or PIP2.** Ten day old seedlings were incubated with 1 µM peptide for 0.5, 1 or 3 h before harvesting the RNA. At least two repeats were performed with similar results.
(TIF)

Figure S7 **Peptide-induced immune activation in roots.** (A) Peptide-induced *MYB51p::GUS* activity in the root. Transgenic seedlings carrying *MYB51p::GUS* incubated with 1 µM peptide for 2 h before GUS staining. (B) Peptide-induced callose deposition in roots. Callose deposits were stained after a 16 h induction with 1 µM peptide. At least two repeats were performed with similar results.
(TIF)

Figure S8 **Fluorescence microscopy image of** ***A. thaliana*** **roots infected with** ***Foc*** **699-** ***GFP*** **.** (A) The primary root after co-cultivation with *Foc* 699-*GFP* for 24 h. (B) The elongation zone of primary root after co-cultivation with *Foc* 699-*GFP*. At least two repeats were performed with similar results.
(TIF)

Figure S9 **Root growth inhibition by PIP1 and PIP2.** (A) T-DNA insertion sites in the *rlk7* and *hsl3* mutants with exons shown as black boxes (top and middle). Primers indicated by LP and RP were used to identify the *RLK7* and *HSL3* transcripts. RT-PCR analysis of *RLK7*, *HSL3* and *Actin2* (control) transcripts in Col-0 and T-DNA insertion mutants of *RLK7* and *HSL3* (bottom). (B) Morphology and (C) root length of eight day old *A. thaliana* WT and *rlk7-2* mutant seedlings in the presence of 1 µM PIP1. (D) Morphology and (E) root length of eight day old *A. thaliana* WT and *rlk7-3* mutant seedlings in the presence of 1 µM PIP2. (C) and (E) Means marked by “a” differed significantly (p<0.01) from those marked “b” (*t*-test). At least two repeats were performed with similar results.
(TIF)

Figure S10 **PIP1 and PIP2-induced responses in RLK7-dependent.** PIP1- and PIP2-induced transcription of (A) *WRKY33* and *WRKY53*, and (B) *FRK1* in WT, *rlk7-3* and *hsl3-1* mutants. (C) flg22-induced expression of *FRK1* in WT, *fls2* and *rlk7-3* mutants. Ten day old seedlings were incubated with 1 µM peptide for 0.5 (*WRKY33* and *WRKY53*) or 3 h (*FRK1*) before harvesting the RNA. At least two repeats were performed with similar results.
(TIF)

Figure S11 **Activity detection of biotinylated PIP1.** (A) Root growth inhibition induced by biotin-PIP1 and PIP1-biotin. (B) *WRKY33* and *WRKY53* expression induced by PIP1, biotin-PIP1 and PIP1-biotin. Statistically significant (p<0.01) differences were indicated by different letters (*t*-test). Two repeats were performed with similar results.
(TIF)

Figure S12 **PEP1 activities in** ***rlk7*** **.** (A) Root growth inhibition induced by PIP1 and PEP1 in WT and *rlk7-2*. (B) *WRKY33* expression induced by PIP1 and PEP1 in WT and *rlk7-2*. Statistically significant (p<0.01) differences were indicated by different letters (*t*-test). Two repeats were performed with similar results.
(TIF)

Table S1 **Secreted peptide precursor genes in** ***A. thaliana*** **up-regulated (≥2 fold) by PAMP treatments.** ^a^The data were obtained from a microarray analysis (microarray accession number E-MEXP-547). ^b^elf18 represents the active epitope of EF-Tu form *Agrobacterium tumefaciens*.
(DOC)

Table S2 **Peptide sequences used in this study.** P(OH) and Hyp represent Hydroxyproline.
(DOC)

Table S3 **Oligonucleotide sequences used in this study.**
(DOC)
