# Correction: The Secreted Peptide PIP1 Amplifies Immunity through Receptor-Like Kinase 7

**DOI:** 10.1371/journal.ppat.1004710

**Published:** 2015-02-19

**Authors:** The PLOS Pathogens Staff

There is an error in the following affiliation for authors Shuguo Hou, Xin Wang, Donghua Chen, Xue Yang, Mei Wang, Wei Zhang:

School of Life Science, Shandong University, Jinan, Shandong, China

The correct affiliation is:

Key Laboratory of Plant Cell Engineering and Germplasm Innovation, Ministry of Education, School of Life Science, Shandong University, Jinan, Shandong, China
