# Supplementary figures and images for: The Secreted Peptide PIP1 Amplifies Immunity through Receptor-Like Kinase 7
Source: PLoS Pathog. 2014 Sep 4;10(9):e1004331. doi: 10.1371/journal.ppat.1004331 (PMC4154866; doi:10.1371/journal.ppat.1004331)

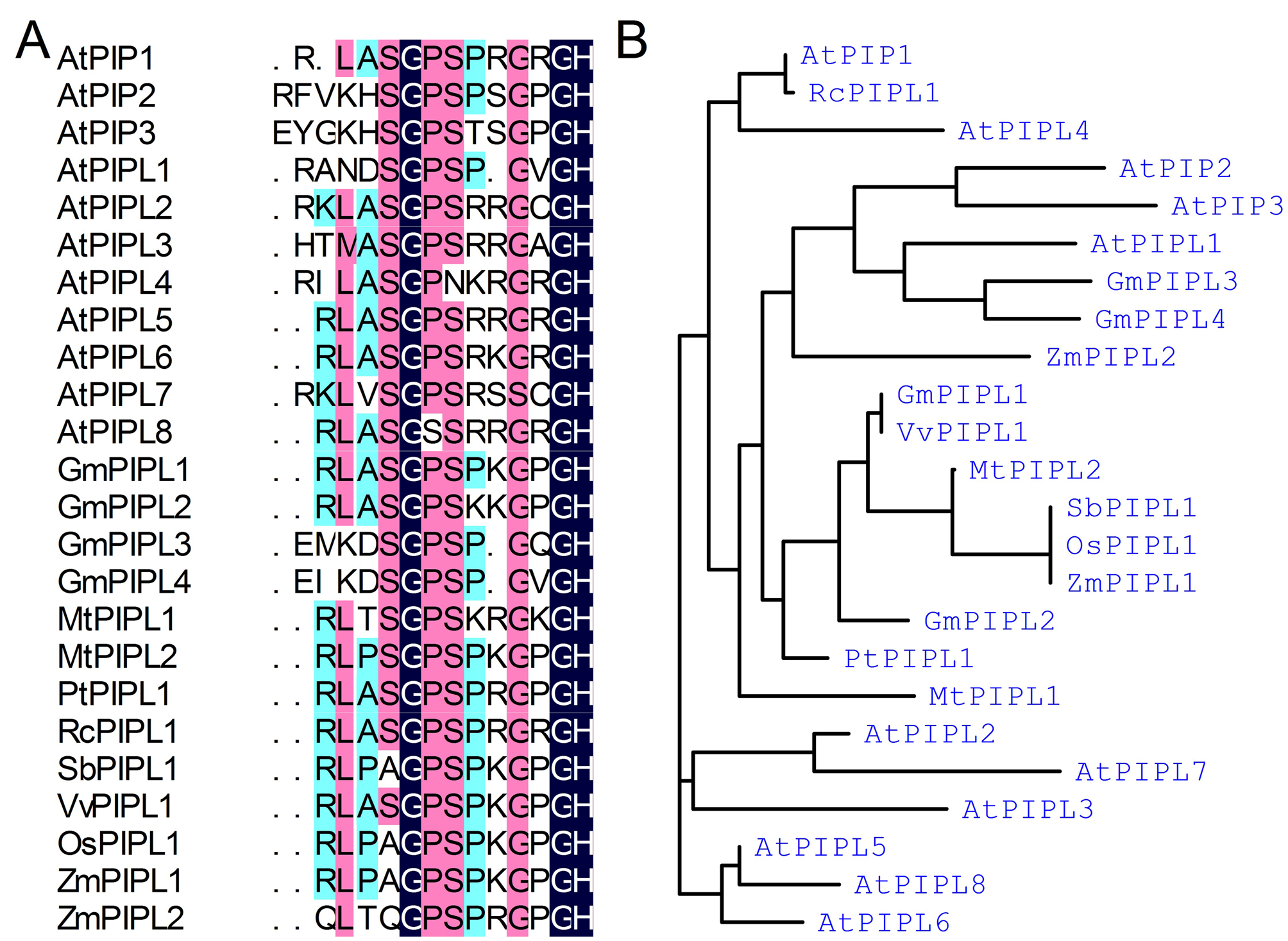

Supplement: Figure S1 — SGPS-motif of prePIP homologs in various plants. (A) Multiple sequence alignments of the conserved C-termini in prePIP homologs. (B) A neighbor-joining phylogenetic tree of the C-terminal sequences in prePIP homologs. GenBank accession numbers are as follows: ACU15907 (GmPIPL1), NP_001238364 (GmPIPL2), XP_006606893 (GmPIPL3), NP_001239759 (GmPIPL4), ACG48199 (ZmPIPL1), ACG26477 (ZmPIPL2), NP_001175941 (OsPIPL1), XP_003632092 (VvPIPL1), XP_003589124 (MtPIPL1), XP_003606833 (MtPIPL2), XP_002534518 (RcPIPL1), XP_002322914 (PtPIPL1), XP_002462659 (SbPIPL1). (TIF) [file ppat.1004331.s001.tif]

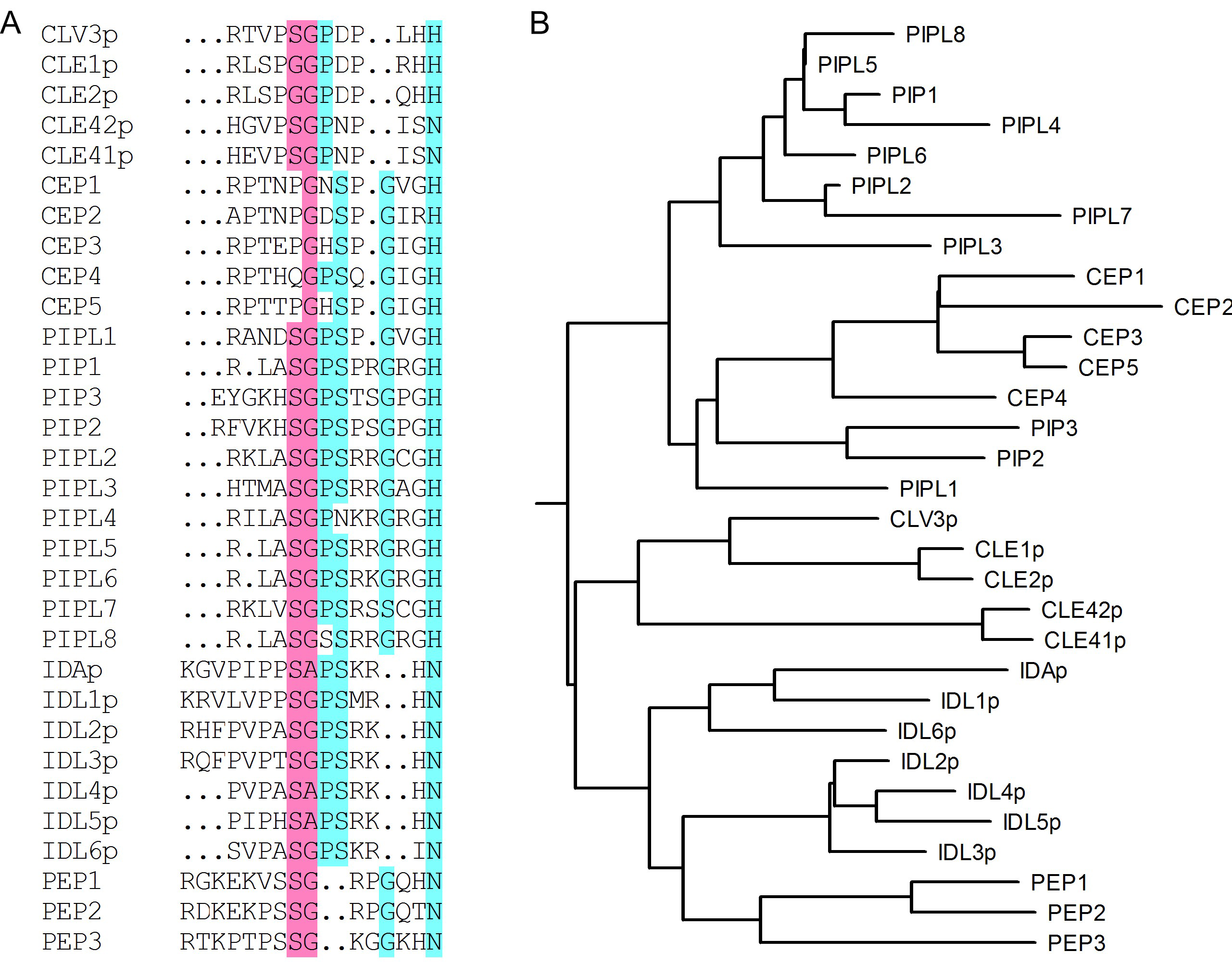

Supplement: Figure S2 — A. thaliana SGP-rich peptide sequences. (A) Multiple sequence alignment. (B) A neighbor-joining phylogenetic tree. (TIF) [file ppat.1004331.s002.tif]

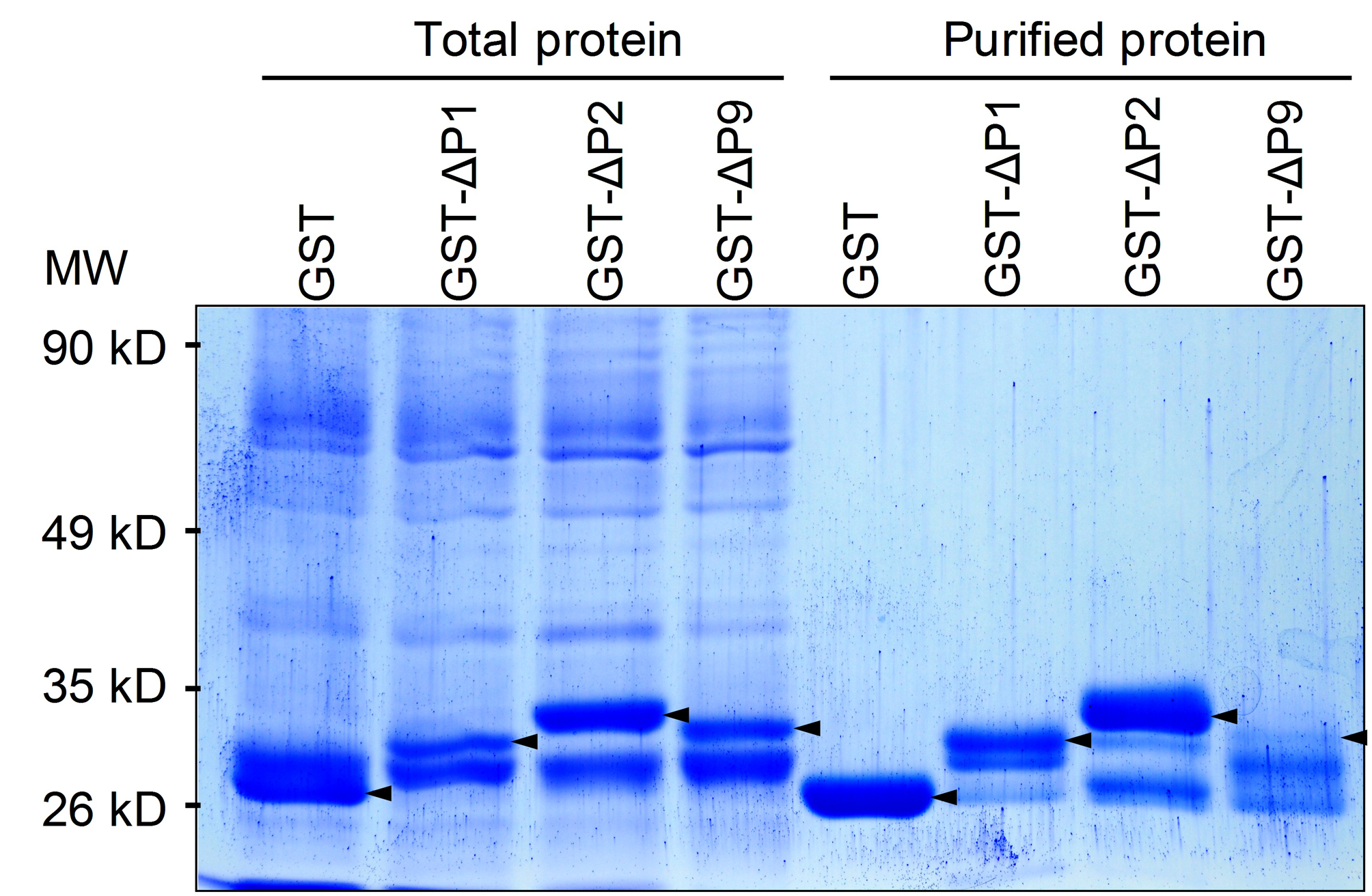

Supplement: Figure S3 — Expression and purification of GST and GST-ΔprePIPs (GST-ΔPs) from E. coli strain BL21 (DE3). Proteins were separated by SDS-PAGE and detected using Coomassie Brilliant Blue staining. Arrows mark the expressed GST and GST-ΔPs. (TIF) [file ppat.1004331.s003.tif]

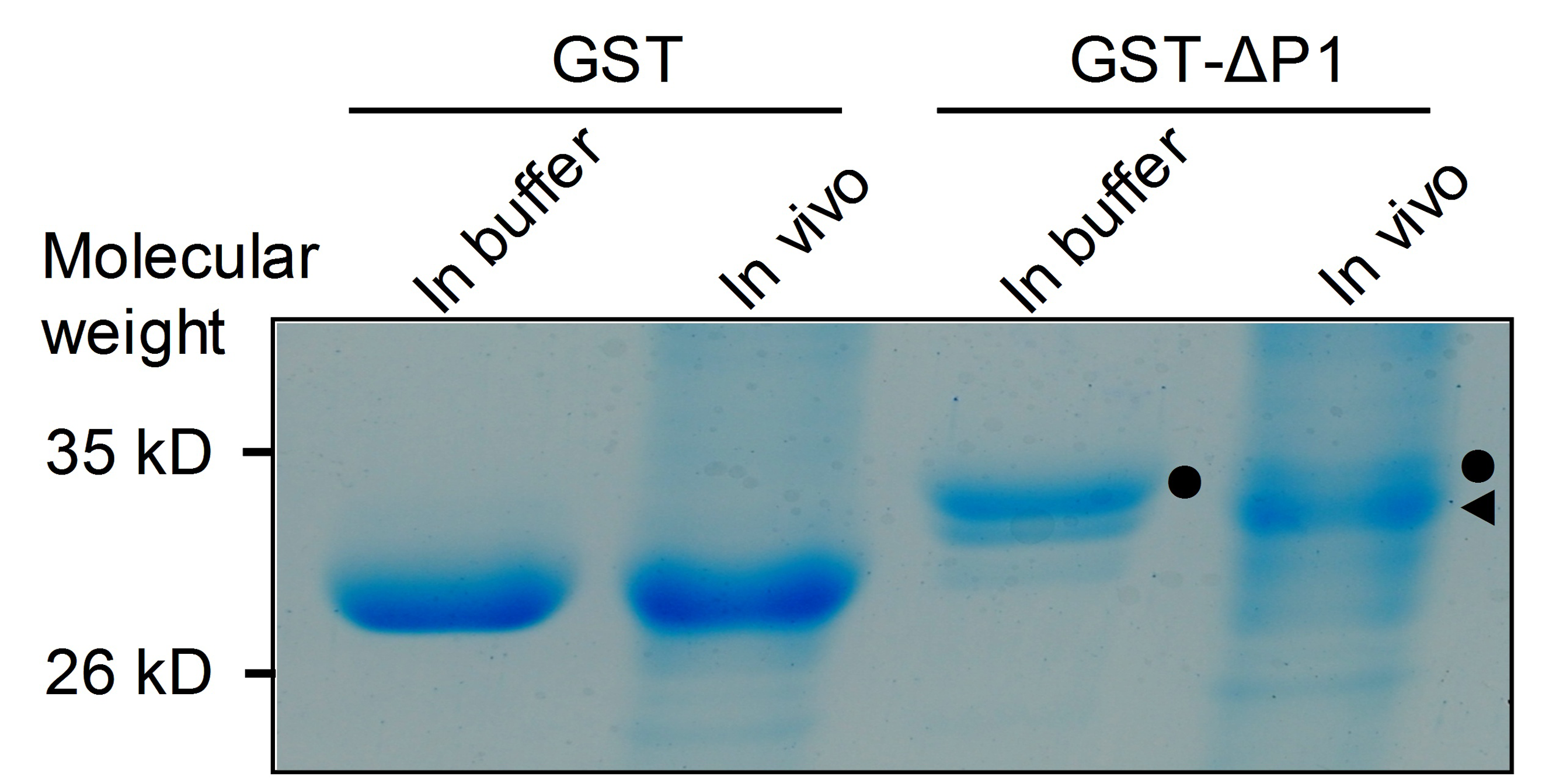

Supplement: Figure S4 — GST-ΔP1 cleavage in vivo . GST-ΔP1 or GST (control) was injected into A. thaliana leaves. Extracellular fruit was extracted for SDS-PAGE detection. Dots mark intact GST-ΔP1, triangles processed GST-ΔP1. Two repeats were performed with similar results. (TIF) [file ppat.1004331.s004.tif]

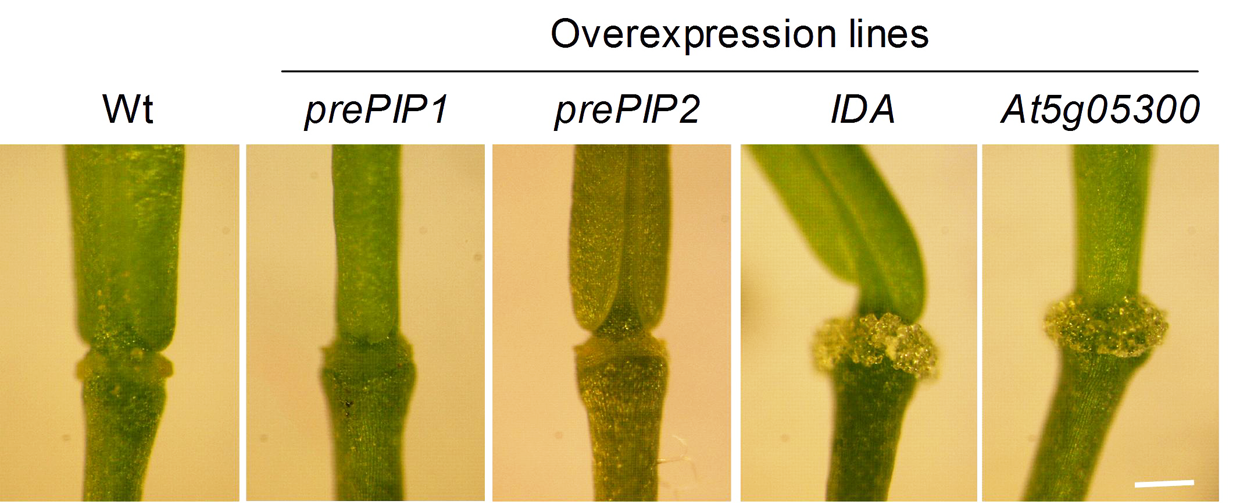

Supplement: Figure S5 — The floral abscission region of A. thaliana over-expressing prePIP1 , prePIP2 , IDA and At5g05300 (bar = 1 mm). (TIF) [file ppat.1004331.s005.tif]

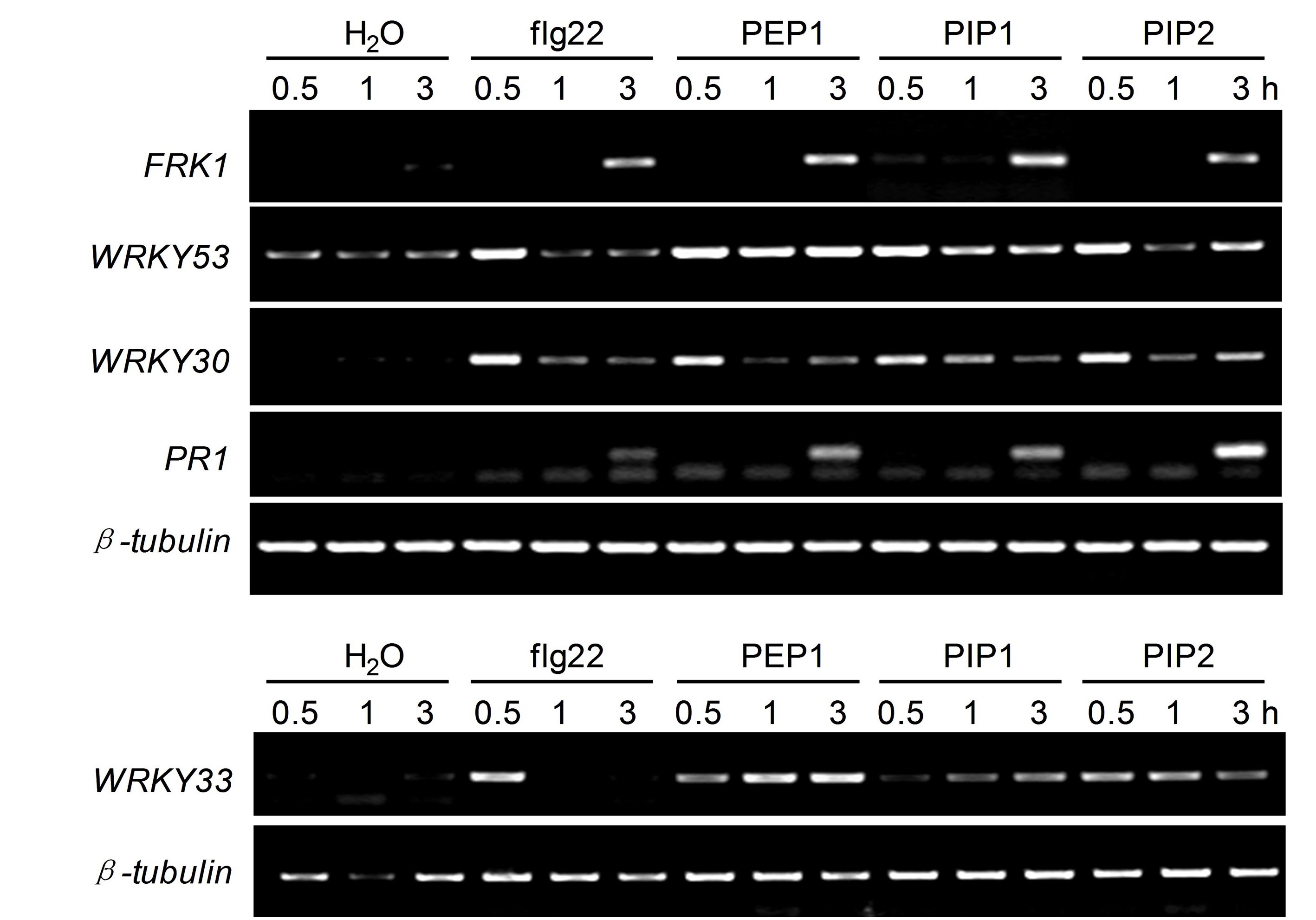

Supplement: Figure S6 — Transcript abundance of FRK1 , WRKY33 , WRKY53 , and PR1 upon induction with flg22, PEP1, PIP1 or PIP2. Ten day old seedlings were incubated with 1 µM peptide for 0.5, 1 or 3 h before harvesting the RNA. At least two repeats were performed with similar results. (TIF) [file ppat.1004331.s006.tif]

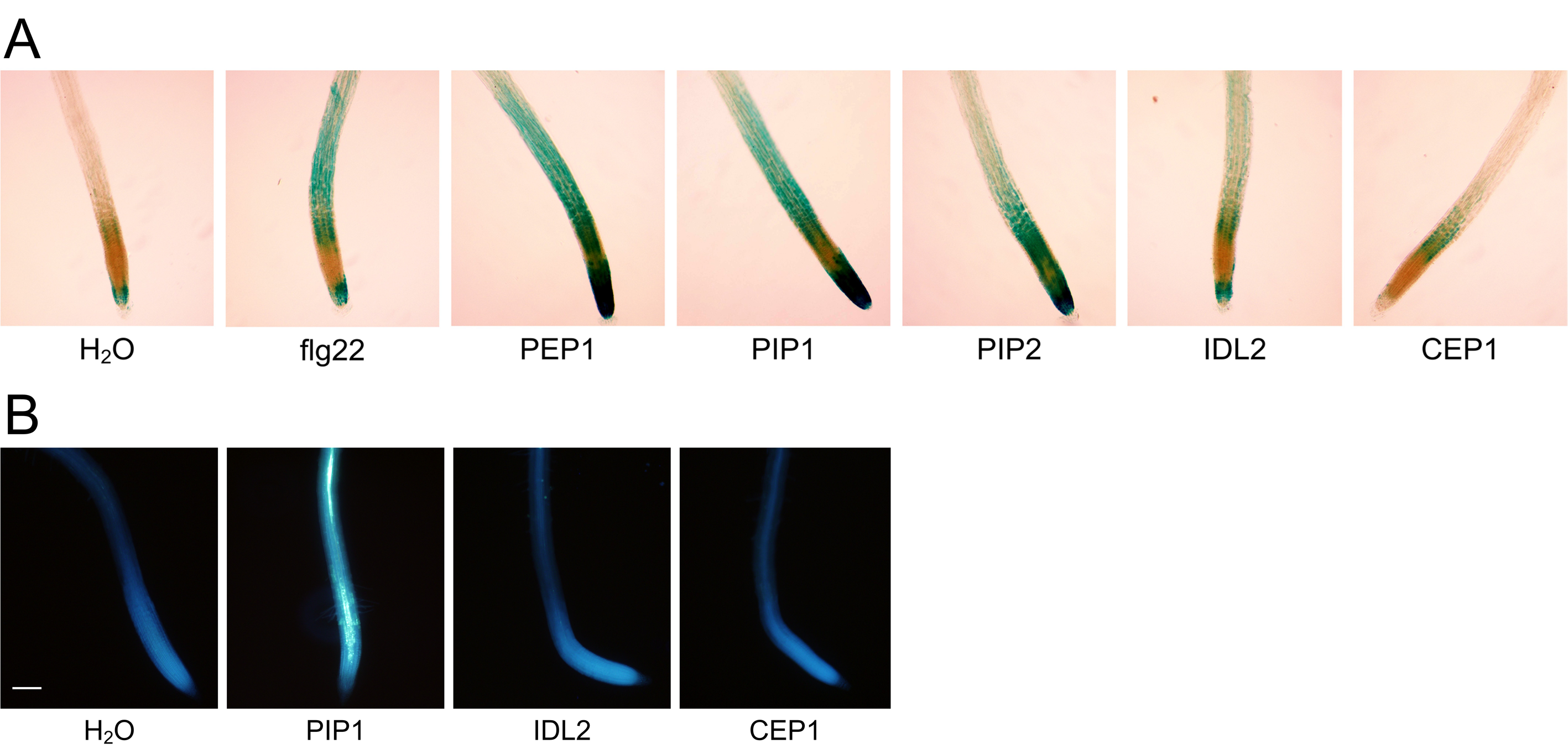

Supplement: Figure S7 — Peptide-induced immune activation in roots. (A) Peptide-induced MYB51p::GUS activity in the root. Transgenic seedlings carrying MYB51p::GUS incubated with 1 µM peptide for 2 h before GUS staining. (B) Peptide-induced callose deposition in roots. Callose deposits were stained after a 16 h induction with 1 µM peptide. At least two repeats were performed with similar results. (TIF) [file ppat.1004331.s007.tif]

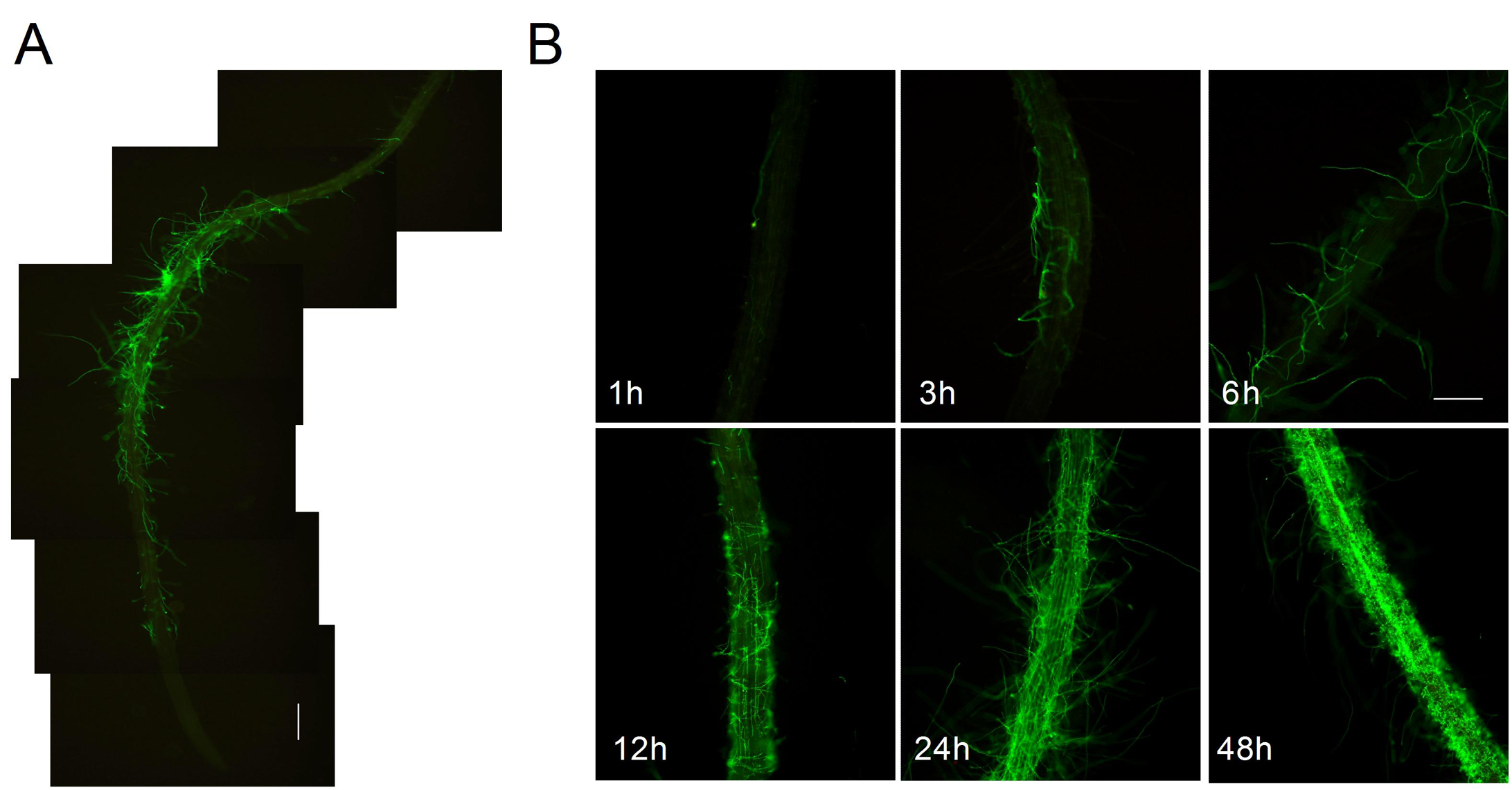

Supplement: Figure S8 — Fluorescence microscopy image of A. thaliana roots infected with Foc 699- GFP . (A) The primary root after co-cultivation with Foc 699-GFP for 24 h. (B) The elongation zone of primary root after co-cultivation with Foc 699-GFP. At least two repeats were performed with similar results. (TIF) [file ppat.1004331.s008.tif]

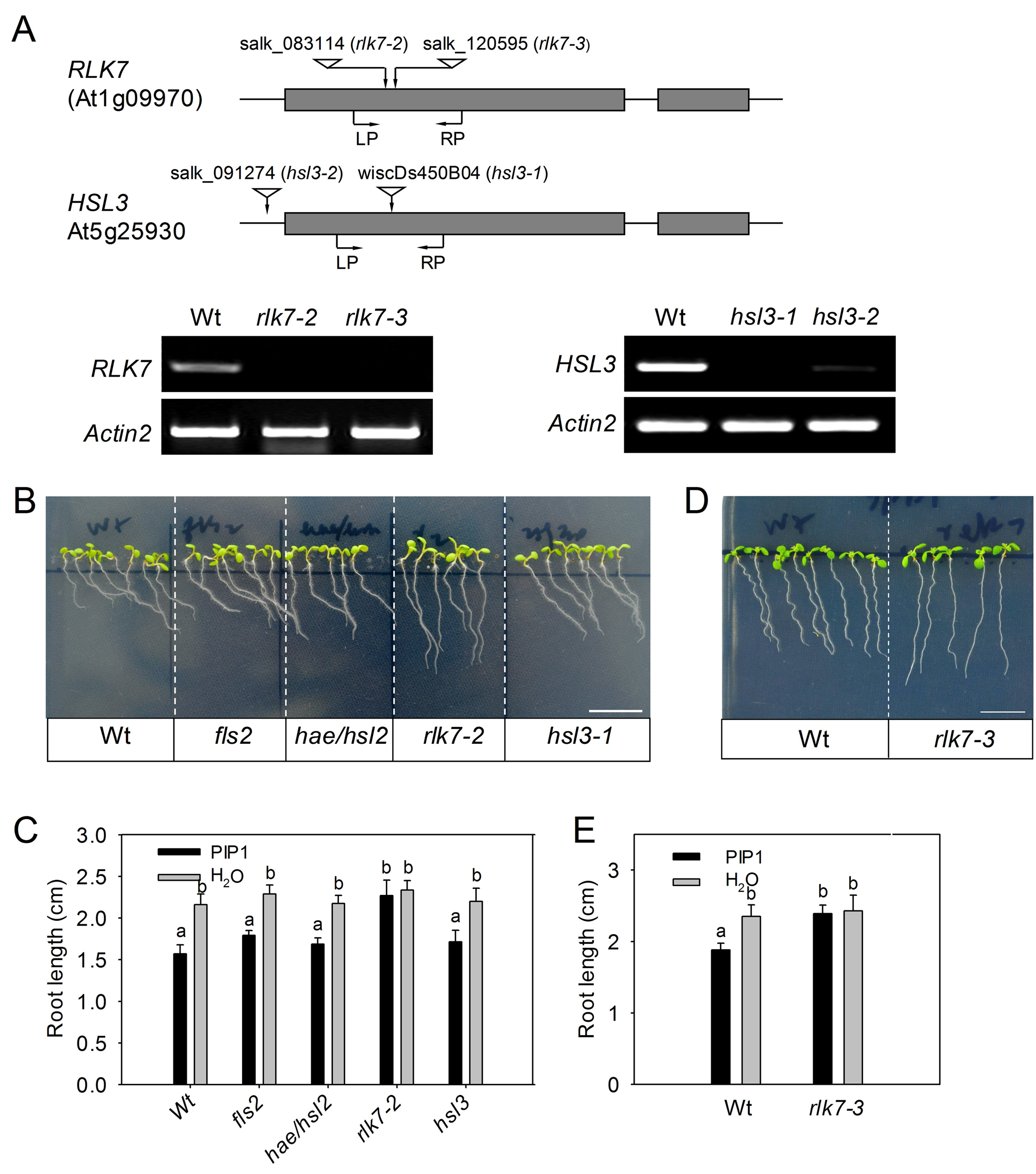

Supplement: Figure S9 — Root growth inhibition by PIP1 and PIP2. (A) T-DNA insertion sites in the rlk7 and hsl3 mutants with exons shown as black boxes (top and middle). Primers indicated by LP and RP were used to identify the RLK7 and HSL3 transcripts. RT-PCR analysis of RLK7, HSL3 and Actin2 (control) transcripts in Col-0 and T-DNA insertion mutants of RLK7 and HSL3 (bottom). (B) Morphology and (C) root length of eight day old A. thaliana WT and rlk7-2 mutant seedlings in the presence of 1 µM PIP1. (D) Morphology and (E) root length of eight day old A. thaliana WT and rlk7-3 mutant seedlings in the presence of 1 µM PIP2. (C) and (E) Means marked by “a” differed significantly (p<0.01) from those marked “b” (t-test). At least two repeats were performed with similar results. (TIF) [file ppat.1004331.s009.tif]

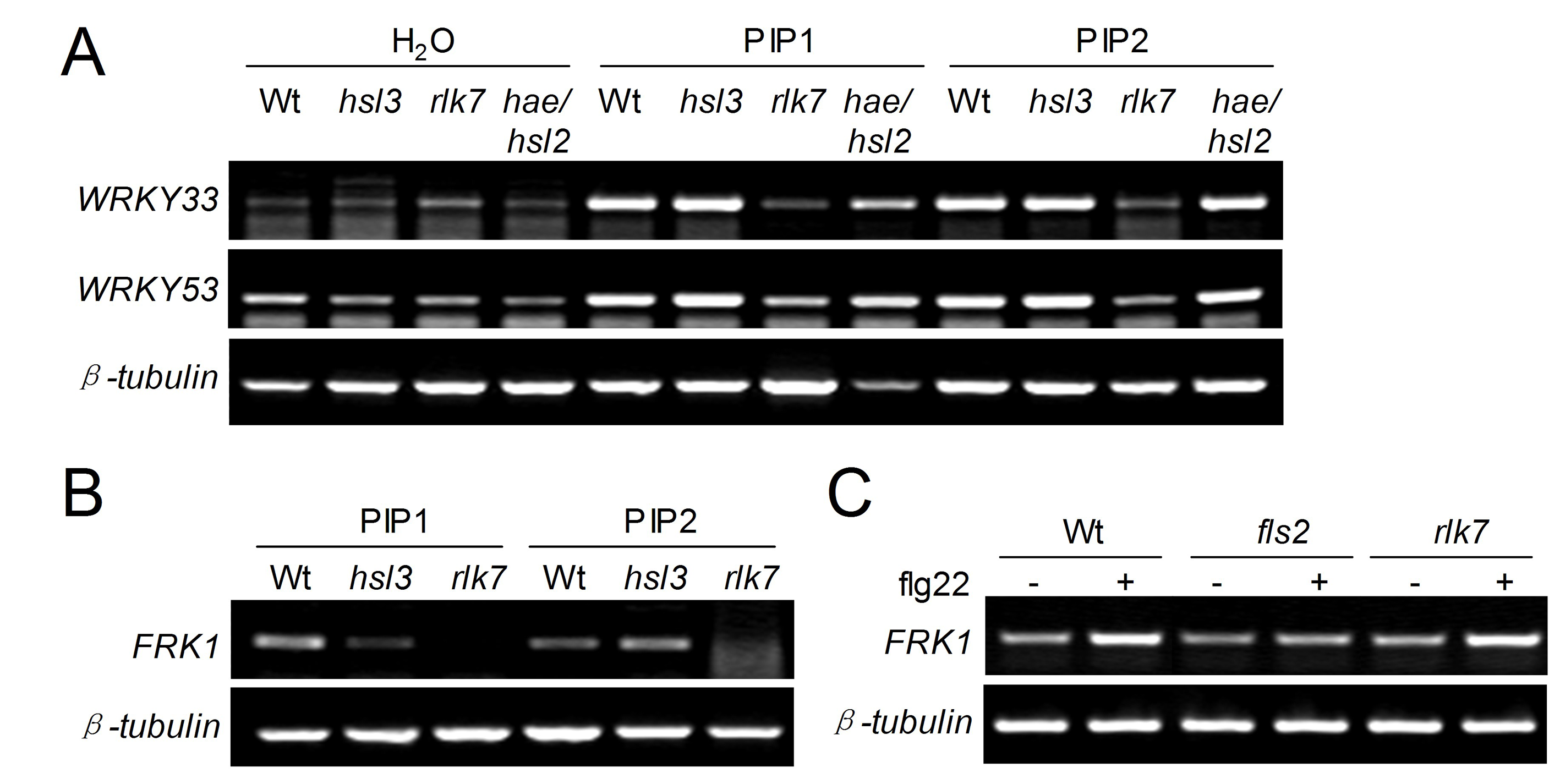

Supplement: Figure S10 — PIP1 and PIP2-induced responses in RLK7-dependent. PIP1- and PIP2-induced transcription of (A) WRKY33 and WRKY53, and (B) FRK1 in WT, rlk7-3 and hsl3-1 mutants. (C) flg22-induced expression of FRK1 in WT, fls2 and rlk7-3 mutants. Ten day old seedlings were incubated with 1 µM peptide for 0.5 (WRKY33 and WRKY53) or 3 h (FRK1) before harvesting the RNA. At least two repeats were performed with similar results. (TIF) [file ppat.1004331.s010.tif]

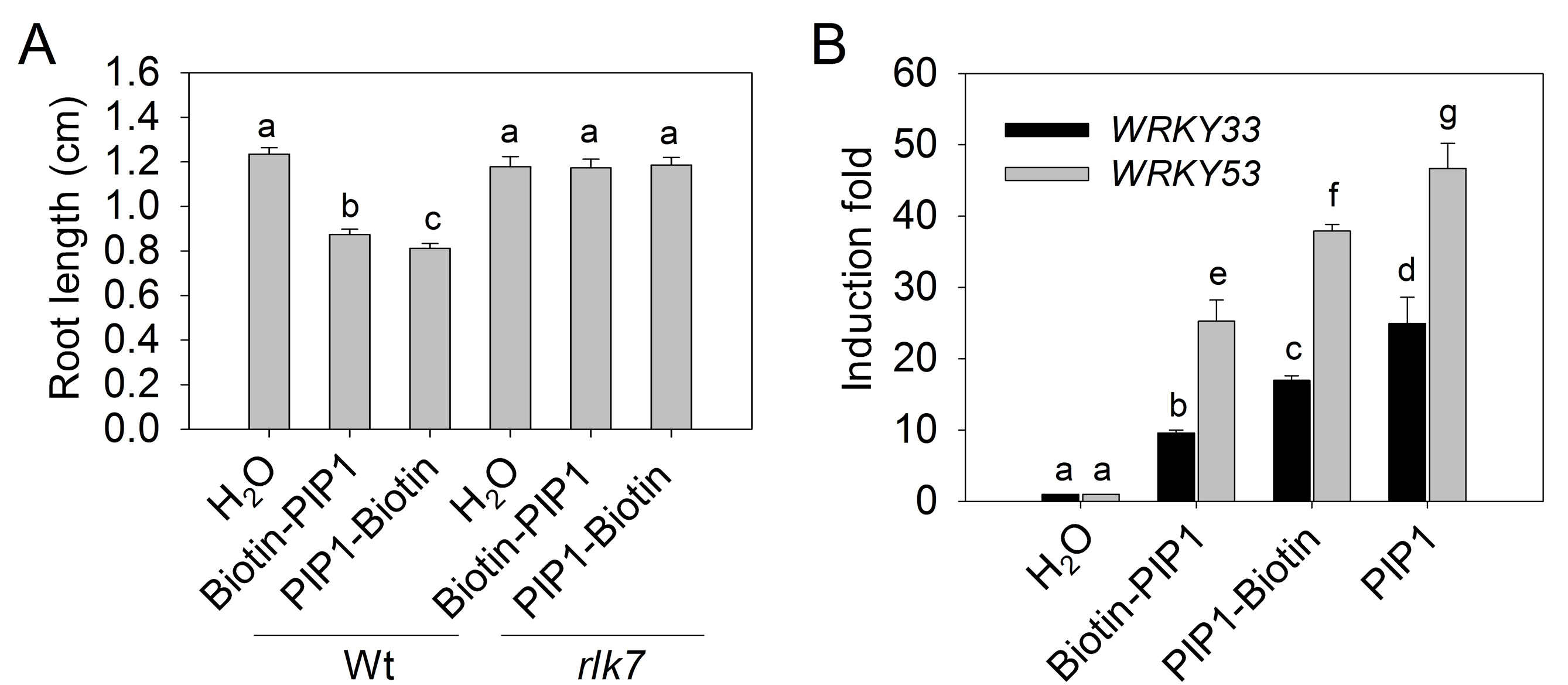

Supplement: Figure S11 — Activity detection of biotinylated PIP1. (A) Root growth inhibition induced by biotin-PIP1 and PIP1-biotin. (B) WRKY33 and WRKY53 expression induced by PIP1, biotin-PIP1 and PIP1-biotin. Statistically significant (p<0.01) differences were indicated by different letters (t-test). Two repeats were performed with similar results. (TIF) [file ppat.1004331.s011.tif]

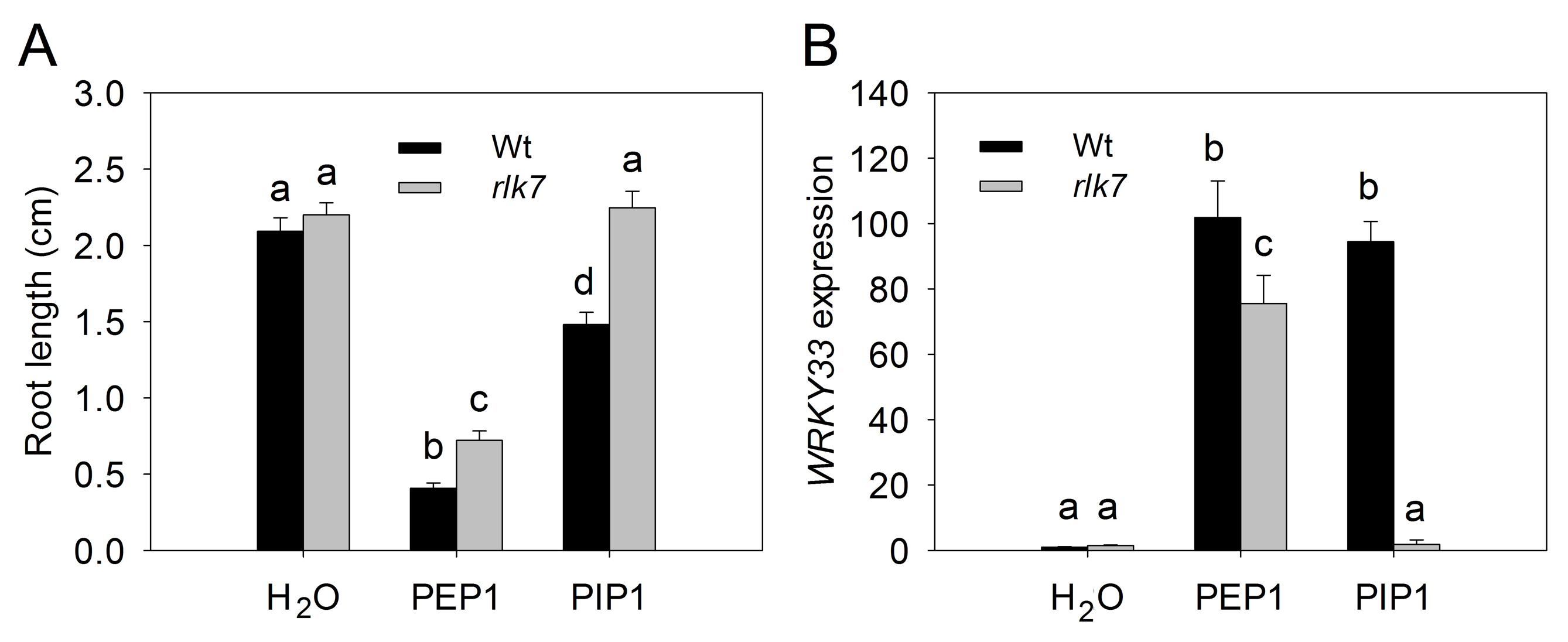

Supplement: Figure S12 — PEP1 activities in rlk7 . (A) Root growth inhibition induced by PIP1 and PEP1 in WT and rlk7-2. (B) WRKY33 expression induced by PIP1 and PEP1 in WT and rlk7-2. Statistically significant (p<0.01) differences were indicated by different letters (t-test). Two repeats were performed with similar results. (TIF) [file ppat.1004331.s012.tif]
