# Supplementary material for: The Secreted Peptide PIP1 Amplifies Immunity through Receptor-Like Kinase 7
Source: PLoS Pathog. 2014 Sep 4;10(9):e1004331. doi: 10.1371/journal.ppat.1004331 (PMC4154866; doi:10.1371/journal.ppat.1004331)
Supplement: Table S1 — Secreted peptide precursor genes in A. thaliana up-regulated (≥2 fold) by PAMP treatments. aThe data were obtained from a microarray analysis (microarray accession number E-MEXP-547). belf18 represents the active epitope of EF-Tu form Agrobacterium tumefaciens. (DOC) [file ppat.1004331.s013.doc]

| Code | Locus | Names or Description | Length (amino acids) | Functions | Induction Fold (30 min)^a^ | |
| --- | --- | --- | --- | --- | --- | --- |
|  |  |  |  |  | flg22 | elf18^b^ |
| 1 | At5g58650 | PSY1 precursor | 75 | Development and immunity | 2.2 | 1.6 |
| 2 | At3g49780 | PSK4 precursor | 79 | Development and immunity | 2.4 | 2.4 |
| 3 | At1g68765 | IDA | 77 | Floral organ abscission | 2.2 | 6.9 |
| 4 | At5g05300 | IDA-like protein | 102 | Floral organ abscission | 18.8 | 25 |
| 5 | At4g28460 | Unknown protein | 72 | Unknown | 26.2 | 44.1 |
| 6 | At4g37290 | Unknown protein | 84 | Unknown | 11.0 | 6.9 |
| 7 | At2g23270 | Unknown protein | 86 | Unknown | 9.5 | 10.1 |
| 8 | At4g28085 | Unknown protein | 84 | Unknown | 5.0 | 9.4 |
| 9 | At1g22890 | Unknown protein | 73 | Unknown | 4.0 | 4.4 |
| 10 | At1g36640 | Unknown protein | 75 | Unknown | 3.7 | 7.5 |
| 11 | At5g12880 | Unknown protein | 73 | Unknown | 2.6 | 7.9 |
| 12 | At1g51920 | Unknown protein | 78 | Unknown | 1.9 | 5.6 |
