# Supplementary material for: The Secreted Peptide PIP1 Amplifies Immunity through Receptor-Like Kinase 7
Source: PLoS Pathog. 2014 Sep 4;10(9):e1004331. doi: 10.1371/journal.ppat.1004331 (PMC4154866; doi:10.1371/journal.ppat.1004331)
Supplement: Table S2 — Peptide sequences used in this study. P(OH) and Hyp represent Hydroxyproline. (DOC) [file ppat.1004331.s014.doc]

| Name | Sequence (from N terminus to C terminus) |
| --- | --- |
| flg22 | QRLSTGSRINSAKDDAAGLQIA |
| PEP1 | ATKVKAKQRGKEKVSSGRPGQHN |
| PIP1 and PIP1^Hyp6^ | RLASGP(OH)SPRGPGH |
| PIP2 | RFVKHSGP(OH)SPSGPGH |
| PIP1^0^ | RLASGPSPRGPGH |
| PIP1^Hyp8^ | RLASGPSP(OH)RGPGH |
| PIP1^Hyp6, 8^ | RLASGP(OH)SP(OH)RGPGH |
| Biotin-PIP1 | Biotin-RLASGP(OH)SPRGPGH |
| PIP1-Biotin | RLASGP(OH)SPRGPGH-K-biotin |
| Y-PIP1 | YRLASGP(OH)SPRGPGH |
| IDL2 | RHFPVPASGPSRKHN |
| CEP1 | DFRP(OH)TNPGNSP(OH)GVGH |
