# Supplementary material for: The Secreted Peptide PIP1 Amplifies Immunity through Receptor-Like Kinase 7
Source: PLoS Pathog. 2014 Sep 4;10(9):e1004331. doi: 10.1371/journal.ppat.1004331 (PMC4154866; doi:10.1371/journal.ppat.1004331)
Supplement: Table S3 — Oligonucleotide sequences used in this study. (DOC) [file ppat.1004331.s015.doc]

| **Gene, Mutant, and Vector** | **Primer Sequence (5’-3’)** | | | **Use** |
| --- | --- | --- | --- | --- |
|  | **Forward (or LP)** | **Reverse (or RP)** | **BP Primer or Restriction site** |  |
| *WRKY53* (At4g23810) | GAAGAGTTTGCCGATGGAG | CGAGGCTAATGGTGGTGTT |  | RT-PCR |
| *WRKY53* (At4g23810) | GCGACAAGACACCAGAGACA | ACCGTTGGATTGAACCAGTC |  | qRT-PCR |
| *WRKY33* (At2g38470) | CCTTCTCTTGTCTCTCCTTCC | TCCTTTCCTTTGCTCTCTTC |  | RT-PCR |
| *WRKY33* (At2g38470) | GAAACAAATGGTGGGAATGG | TGTCGTGTGATGCTCTCTCC |  | qRT-PCR |
| *PR1* (At2g14610) | TGTAGGTGCTCTTGTTCTTCC | CATTAGTATGGCTTCTCGTTCA |  | RT-PCR |
| *PDF1.2* (At5g44420) | TGTTCTCTTTGCTGCTTTCGACGC | TGTGTGCTGGGAAGACATAGTTGC |  | RT-PCR |
| *β-tubulin* (At1g20010) | CAACGCTACTCTGTCTGTCC | TCTGTGAATTCCATCTCGTC |  | RT-PCR |
| *Actin2* (At3g18780) | TCCCTCAGCACATTCCAGCAGAT | AACGATTCCTGGACCTGCCTCATC |  | qRT-PCR |
| *FRK1* (At2g19190) | AACGCTTAGTTTCAGACCG | CCTGTGGATGAGTTGGGGAT |  | RT-PCR |
| *FRK1* (At2g19190) | GGAAGCGGTCAGATTTCAAC | AGCTTGCAATAGCAGGTTGG |  | qRT-PCR |
| *WRKY30* (At5g24110) | TTGACCAAGTTTCTCAGGG | TGTTTAGTGGCTTCACATCC |  | RT-PCR |
| *RLK7* (At1g09970) | ACCGATAGAGGCAGAGTTTGG | TGTCCACAATCTCCATCACACT |  | qRT-PCR |
| *PEPR1* (At1g73080) | CAACAACAATGTGGAGGATA | AACGAGATTACCGAACTGAA |  | qRT-PCR |
| *FLS2* (At5g46330) | ACTCTCCTCCAGGGGCTAAGGAT | AGCTAACAGCTCTCCAGGGATGG |  | qRT-PCR |
| *proPEP1* (At5g64900) | ATGGAGAAATCAGATAGACG | ATTATGTTGGCCAGGACG |  | RT-PCR |
| *proPEP1* (At5g64900) | GGATTCCTCTTCAGTGCCTCG | TGCTTTGCCTTGACCTTTGTG |  | qRT-PCR |
| *prePIP1* (At4g28460) | ATGGCCGCGACCTCTTGG | CGATGAGAAGAGTTAGTTGG |  | RT-PCR |
| *prePIP1* (At4g28460) | AATCGGGAGAATGGAAGTGC | GACGCCAAACGCTGAAAC |  | qRT-PCR |
| *prePIP2* (At4g37290) | GTGGCCCGGTCCGCTTGG | CTGCCAAAGAGAAAGAAAACC |  | RT-PCR |
| *prePIP2* (At4g37290) | GGCTAGTTTATTCAGTGGCTTAT | CCTTCCTGTCCACGACCTTA |  | qRT-PCR |
| *sGFP* | TGGAAGCGTTCAACTAGCAG | AAAGGGCAGATTGTGTGGAC |  | qPCR |
| *RLK7* | ACGATTTGATCGTCGTGCTAC | TTACAACAACTCCTTGACCGG |  | RT-PCR |
| *rlk7-2* (salk_083114) | ACGATTTGATCGTCGTGCTAC | TTACAACAACTCCTTGACCGG | AACGTCCGCAATGT GTTATTAAGTTGTC | Genotyping |
| *rlk7-3* (salk_120595) | ACGATTTGATCGTCGTGCTAC | TTACAACAACTCCTTGACCGG |  | Genotyping |
| *fls2* (salk_026801C) | TCCTGATCTGCCTGCAATAAG | GTTGGAGCAAGCAACAGATTC |  | Genotyping |
| *bak1-4* (salk_116202) | CATGACATCATCATCATTCGC | ATTTTGCAGTTTTGCCAACAC |  | Genotyping |
| *hsl3-1* (wiscDsLox450B04) | TGCTCTGGTATTCCTCCAGTG | ATCCCAGATGTTTTATTCGGG | ATTTTGCCGATTT  CGGAAC | Genotyping |
| *pCAMBIA1300-prePIP1-HA* | ATGGATCCATGAGAAGAGTTAGTTGGTC | ATAAGCTTATGGCCGCGACCTCTTGG | Kpn I/Hind III | Construction |
| *pCAMBIA1300-prePIP2-HA* | ATGGATCCATGATGATGAACAAAAACG | ACAAGCTTGTGGCCCGGTCCGCTTGG | Kpn I/Hind III | Construction |
| *pCAMBIA1300-RLK7-HA* | CAGGTACCACTCAAACCACCGTCACCACT | TTGTCGACTTCTTTGACCTTGACATCACTCTC | Kpn I/Sal I | Construction |
| *prePIP1p::GUS* and *prePIP1p::GFP* | CCCATGGGAGAGACACAGAGAGATACAGAG | CCCATGGCTCTTCTTTATGTTGTACCGAC | Nco I/ Nco I | Construction |
| *pCAMBIA1300-prePIP1-GFP* | ACTCTAGAACGATGAGAAGAGTTAGTTG | ATGGTACCATGGCCGCGACCTCTTGG | Xba I/Kpn I | Construction |
| *pCAMBIA1300-CLV3-GFP* | ACTCTAGAATGGATTCGAAGAGTTTTCTG | ACGGTACCGTTGTTTCTTGGCTGTCTTG | Xba I/Kpn I | Construction |
| *pGEX-GST::ΔprePIP1* | GGAATTCCTTACTGAAAAATCGGGAG | ACCTCGAGGTTTTCACCGTTTGTTCTGG | EcoR I/Xho I | Construction |
| *pGEX-GST::ΔprePIP2* | GGAATTCCTAACAAAGACCGAGGAGAAG | CACTCGAGTGGGTATGGGCACTTTAAC | EcoR I/Xho I | Construction |
| *pGEX-GST::ΔprePIPL5* | CGGAATTCCGAACAGAGCAAACGACC | TAGCTCGAGCTAGTGTCCACGGCCTTTC | EcoR I/Xho I | Construction |
| *pGBKT7-BIK1* | AAGAATTCTGCTTCAGTTCTCGAGTCAAAG | AACTGCAGCTACACAAGGTGCCTGCCA | EcoR I/Pst I | Construction |
| *pGADT7-PEPR1_KD_* | GTGAATTCCTAAATGAAAAGTACACCATTG | AACTCGAGCCTCTGATTCAGTTCGGTAA | EcoR I/Xho I | Construction |
| *pGADT7-RLK7_KD_* | GTGAATTCTTGATTGGTAGAGGAGGTTG | GTGGATCCTCAACTTATTTCTTTGACCTTG | EcoR I/BamH I | Construction |
